# Earliest evidence of smoke-dried mummification: More than 10,000 years ago in southern China and Southeast Asia

**DOI:** 10.1073/pnas.2515103122

**Published:** 2025-09-15

**Authors:** Hsiao-chun Hung, Zhenhua Deng, Yiheng Liu, Zhiyu Ran, Yue Zhang, Zhen Li, Yousuke Kaifu, Qiang Huang, Khanh Trung Kien Nguyen, Hai Dang Le, Guangmao Xie, Anh Tuan Nguyen, Mariko Yamagata, Truman Simanjuntak, Sofwan Noerwidi, Mohammad Ruly Fauzi, Marlin Tolla, Alpius Wetipo, Gang He, Junmei Sawada, Chi Zhang, Peter Bellwood, Hirofumi Matsumura

**Affiliations:** ^a^Department of Archaeology and Natural History, School of Culture, History and Language, College of Asia and the Pacific, Australian National University, Canberra, ACT 2601, Australia; ^b^Key Laboratory of Archaeological Science (Peking University), Ministry of Education, Beijing 100871, China; ^c^School of Archaeology and Museology, Peking University, Beijing 100871, China; ^d^Guangxi Institute of Cultural Relic Protection and Archaeology, Nanning 530003, China; ^e^The University Museum, The University of Tokyo, Tokyo 113-0033, Japan; ^f^The Dingsishan Site Museum, Nanning 530200, China; ^g^Center for Archaeology, Southern Institute for Social Sciences, Ho Chi Minh City 700000, Vietnam; ^h^Vietnam Institute of Archaeology, Academy of Social Science, Hanoi 100000, Vietnam; ^i^School of History, Culture and Tourism, Guangxi Normal University, Guilin 541006, China; ^j^College of Arts, Rikkyo University, Tokyo 171-8501, Japan; ^k^Center for Prehistoric and Austronesian Studies, Jakarta 12710, Indonesia; ^l^Research Center for Archaeometry, The National Research and Innovation Agency, Jakarta 12710, Indonesia; ^m^Department of Culture and Tourism, Jayawijaya Regency, Wamena 99511, Papua, Indonesia; ^n^Institute of Cultural Relics and Archaeology of Hunan, Changsha 410011, China; ^o^Institute of Physical Anthropology, Niigata University of Health and Welfare, Niigata 950-3198, Japan; ^p^School of Archaeology and Anthropology, College of Arts and Social Sciences, Australian National University, Canberra, ACT 2601, Australia; ^q^Department of Anatomy, Sapporo Medical University, Sapporo, Hokkaido 060-8556, Japan

**Keywords:** mummification, prehistory, Southern China, Southeast Asia, New Guinea Highlands

## Abstract

In southern China and Southeast Asia (collectively, Southeastern Asia), Terminal Pleistocene and Early to Middle Holocene (ca. 12,000 to 4,000 cal. BP) hunter-gatherer burials feature tightly crouched or squatting postures, sometimes with indications of post-mortem dismemberment. Such burials contrast strongly with the extended supine burial postures typical of subsequent Neolithic inhumations in these regions. Their contorted postures, often with traces of burning, present interpretive challenges. This study uses multiple techniques, including X-ray diffraction and Fourier-transform infrared spectroscopy, to investigate 54 pre-Neolithic burials from 11 archaeological sites located across Southeastern Asia. The findings confirm that many of these pre-Neolithic flexed and squatting burials were treated by an extended period of smoke-drying over fire, a process of mummification similar to that recorded ethnographically in some Australian and Highland New Guinea societies. Some of the analyzed archaeological samples represent the oldest known instances of such artificial mummification in the world.

## Significance

This finding documents smoke-dried mummification of the dead, mostly in tightly bound crouched postures, from archaeological contexts between 12,000 and 4,000 y old across a vast region encompassing Southeast Asia, southern China, and beyond. The practice continued into the ethnographic record in the New Guinea Highlands and parts of Australia. The oldest of these burials predate the mummification associated with the Chinchorro culture (7,000 cal. BP, northern Chile) and Ancient Egypt (Old Kingdom, cal. 4,500 BP). Our burial samples from Southeastern Asia highlight a remarkably enduring set of cultural beliefs and mortuary practices that persisted for over 10,000 y among hunter-gatherer communities who were related through their craniofacial attributes and genomic affinities to Indigenous New Guinea Highland and Australian populations.

Prior to the spread of Neolithic populations ancestral to the majority of living East and Southeast Asians, a spread that occurred mainly between 5,000 and 3,500 y ago, the previous hunter-gatherer populations of Southeastern Asia followed a complex set of activities for the mortuary treatment of their deceased community members. Despite some regional variations, pre-Neolithic burials in Southeastern Asia feature predominantly flexed, tightly crouched, or squatting burial postures, often with signs of tight binding of the remains (1–6). Most of these burials are primary rather than secondary, and many display evidence for some degree of burning of the bones. Others are described as exhibiting evidence for possible dismemberment or mutilation, presumably post-mortem. Such observations raise interesting questions about mortuary practices in these pre-Neolithic hunter-gatherer societies of Southeastern Asia.

One of these questions relates to the traditional classification of two distinct burial categories as used in the bioanthropological literature. Typically, primary burials involve the direct interment of a deceased individual in the ground shortly after death, without significant post-mortem alteration. In contrast, secondary burials often involve complex processes such as cremation, or rearrangement of loose skeletal elements. However, many of the burials that we discuss in this paper straddle these primary/secondary definitions and challenge conventional paradigms. Questions have persisted about how ancient people in southeastern Asia achieved the distinctive burial postures of the deceased and what specific post-mortem treatments were applied.

**Fig. 1.**
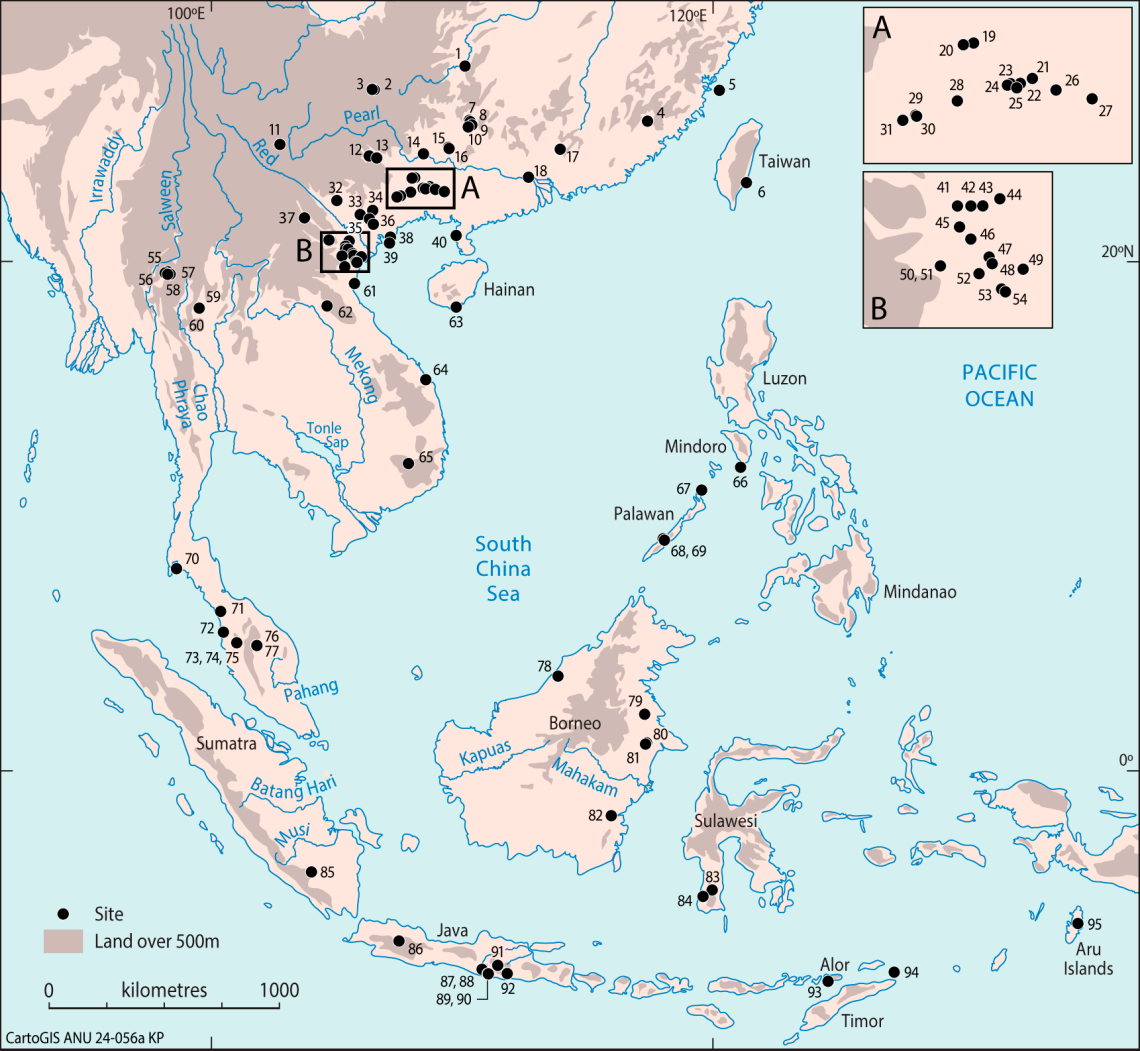
Recorded hunter-gatherer sites with flexed and squatting burials in Southeastern Asia from the Late Pleistocene to the Middle Holocene. 1. Gaomiao, 2. Niupodong, 3. Zhaoguodong, 4. Qihedong*, 5. Daowei 1, 6. Xiaoma, 7. Jiaoziyan, 8. Zengpiyan, 9. Miaoyan, 10. Dayan, 11. Xingyi, 12.Baida, 13. Gexinqiao, 14. Beidaling, 15. Liyuzui, 16. Fengyan, 17. Huangmenyan, 18. Xiankezhou, 19. Baxun, 20. Liyupo, 21. Changtang, 22. Lingwu, 23. Huiyaotian, 24. Qingshan, 25. Dingsishan, 26. Qiujiang, 27. Xijin, 28. Ganzao, 29. Hecun, 30. Jiangbian, 31. Chongtang, 32. Lang Cuom, 33. Mai Da Nguom, 34. Pho Binh Gia, 35. Hang Doi, 36. Dong Thuoc, 37. Hang To 1, 38. Hon Hai Co Tien, 39. Cai Beo, 40. Liyudun, 41. Dong Can, 42. Du Sang, 43. Lang Gao, 44. Hang Cho, 45. Hang Muoi, 46. Xom Trai, 47. Hang Mang Chieng, 48. Hang Con Moong, 49. Hang Diem, 50. Mai Da Dieu, 51. Mai Da Nuoc, 52. Hang Lang Bon, 53. Da But, 54. Con Co Ngua, 55. Banyan Valley Cave, 56. Spirit Cave, 57. Tham Lod, 58. Ban Rai, 59. Doi Pha Kan, 60. Ban Tha Si, 61. Quynh Van, 62. Pha Phen, 63. Yingdun, 64. Bau Du, 65. Krong No, 66. Bubog-1, 67. Ille Cave*, 68. Duyong Cave, 69. Sa’gung, 70. Moh Khiew, 71. Gua Kerbau, 72. Gua Kepah, 73. Gua Kajang, 74. Gua Teluk Kelawar, 75. Gua Gunung Runtuh, 76. Gua Peraling, 77. Gua Cha, 78. Niah Cave, 79. Kimanis, 80. Liang Tebo, 81. Keboboh Cave, 82. Gua Tengkorak, 83. Leang Panninge, 84. Cappalombo1, 85. Gua Harimau, 86. Gua Pawon, 87. Gua Braholo, 88. Song Tritis, 89. Song Terus, 90. Song Keplek, 91. Gua Lawa, 92. Song Gentong, 93. Tron Bon Lei, 94. Ratu Mali 2, 95. Liang Lembudu (*suspected flexed) (see *SI Appendix,* Table S1 for details).

To investigate these questions, we analyzed human bone samples from 95 pre-Neolithic archaeological sites plotted in Fig. 1. Large numbers of these samples come from northern Vietnam and Guangxi Zhuang Autonomous Region in southern China (Fig. 1, *Insets A* and *B*), with smaller numbers reported from the Philippines, Laos, Thailand, Malaysia, and Indonesia. Beyond the boundaries of Fig. 1, such flexed and squatting burials are also reported from hunter-gatherer archaeological contexts in northern China, Korea, Jomon Japan, Australia, and indeed in many regions elsewhere in the world (7–12).

Our research allows us to test a hypothesis that many of our analyzed samples were subjected to long periods of smoke-drying and resulting mummification before burial, similar to ethnographic burial methods that were once widespread in Indigenous Australian societies and that are still practiced today in some regions of the New Guinea Highlands (13–15). To test our hypothesis, we recorded burial positions in the ground and visible burning and cutting marks on excavated bones. Selected samples have been subjected to X-ray diffraction (XRD) and Fourier-transform infrared spectroscopy (FTIR), two laboratory methods used for examining internal bone microstructures. These methods allow detection of the heating of bones by using a low temperature smoking process that might not always have left surface traces of charring visible to the naked eye. Finally, we compare our findings with the ethnographic records of smoke-drying mummification in Australia and the New Guinea Highlands.

## The Burial Samples.

**Fig. 2.**
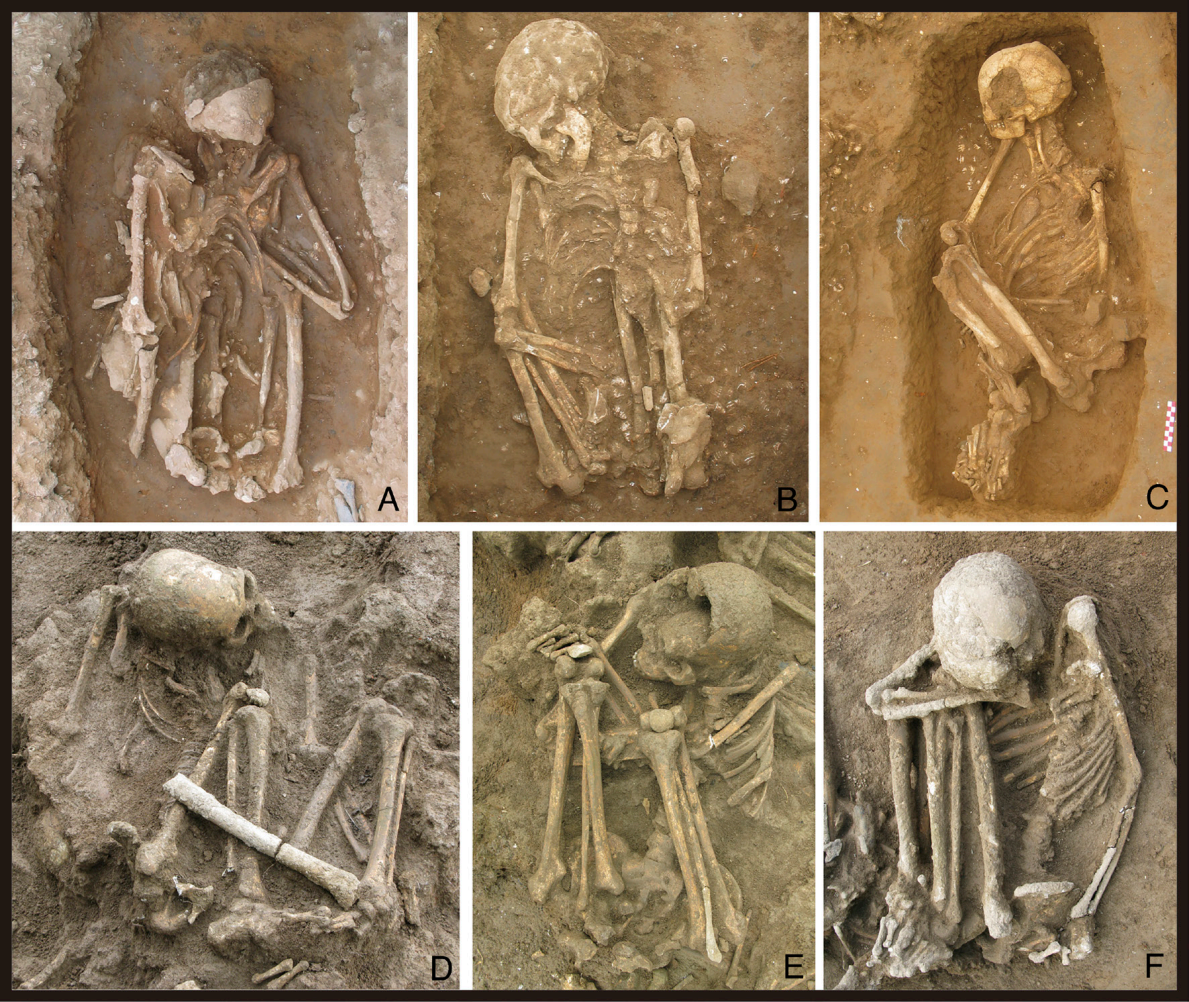
Examples of Early and Middle Holocene human burials from southern China. This figure shows six human burials from Huiyaotian (*A*: M14, *B*: M19, *C*: M20) in Nanning and Liyupo (*D*: M23, *E*: M24, *F*: M28) in Long’an, both shell-midden sites located in the Guangxi Zhuang Autonomous Region. All individuals are in flexed positions, with several exhibiting hyper-flexed postures (*A* and *E*: male, *B*–*D* and *F*: female).

In order to illustrate the contexts in which many of the burials described in this paper were discovered, we describe briefly some of the major cave and open-air sites in Guangxi Zhuang Autonomous Region (southern China), northern Vietnam, and Sumatra (Indonesia) that we have sampled. For instance, Zengpiyan Cave in northeastern Guangxi (occupied ca. 12,000–7,000 calibrated years B.P.) (cal. BP) yielded 26 pre-Neolithic burials, either placed in squatting postures within small pits or positioned in flexed postures lying on their sides (16) (Loc. 8 in Fig. 1 and *SI Appendix,* Fig. S1). In central Guangxi, the shell mound at Huiyaotian on the bank of the Yongjiang River produced 169 burials. A direct radiocarbon date on a human tooth indicates an age of approximately 9,030–8,975 cal. BP (Beta-429237) for this cemetery (Loc. 23 in Fig. 1 and *SI Appendix,* Tables S1 and S2). Among the 60 samples examined from this site, 21 (35%) were flexed and 17 (28%) squatting (Fig. 2 *A*–*C*), the remainder being too poorly preserved for clear observation. Of the better-preserved flexed burials, 12 lay on their sides, three were supine on their backs, and one was prone with its face down (17, 18). At Liyupo, another Guangxi shell mound located about 50 km from Huiyaotian and occupied around 8,000–6,700 cal. BP (Loc. 20 in Fig. 1 and *SI Appendix*, Tables S1 and S2), most burials were flexed and many were covered by large stones placed on their heads, chests or stomachs (19, 20) (Fig. 2 *D*–*F*).

**Fig. 3.**
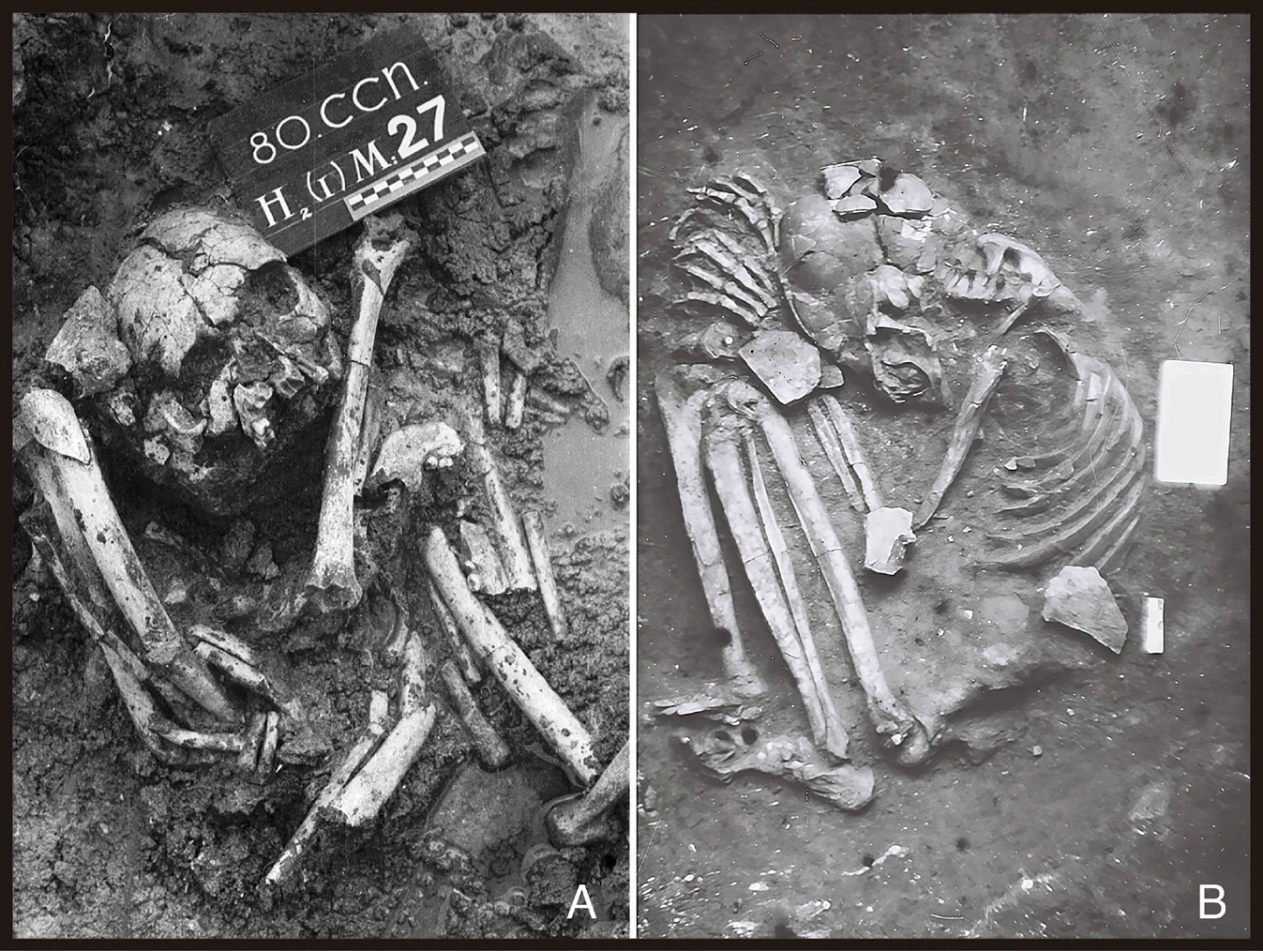
Examples of Early and Middle Holocene flexed burials from northern Vietnam. (*A*) Burial 80M27 from the Con Co Ngua shell midden; (*B*) Burial M16 from Mai Da Dieu Cave, both located in Thanh Hoa Province.

In northern Vietnam, burials of the Hoabinhian-Da But-Quynh Van group, mostly in caves or shell mounds, share similar characteristics with the Guangxi burials in terms of material culture and burial practices. Important sites include Mai Da Dieu, Hang Cho, Hang Diem, Hang Mang Chieng, Con Co Ngua, Quynh Van, and Bau Du (21–26) (Figs. 1 and 3). For instance, the Dabutian coastal shell midden of Con Co Ngua (6,700–6,200 cal. BP) in Thanh Hoa Province yielded 267 burials during excavations in 1978–80 and 2013 (25), predominantly in squatting postures (77%), with 23% flexed on their sides.

Gua Harimau (Loc. 85 in Fig. 1 and *SI Appendix*, Table S2) in southern Sumatra is one of the most significant sites in Island Southeast Asia for examining chronological changes in funerary practices during the Holocene. Intensive excavations in this cave since 2010 have revealed 82 individuals from 33 burial features, most of whom are Late Neolithic and Iron Age individuals interred predominantly in primary extended supine positions or as secondary burials. A smaller number of pre-Neolithic burials exhibit primary flexed postures (4, 5, 27), and two of them are directly C14 dated to 5,715–5,587 cal. BP (Individual 80, Beta-450669) and 3,819–3,568 cal. BP (Individual 79, Beta-452948). The younger end of this date range represents, so far, the latest occurrence of this flexed or squatting burial type in Southeastern Asia.

## Questions Addressed Through the Analyzed Burial Assemblages.

**Fig. 4.**
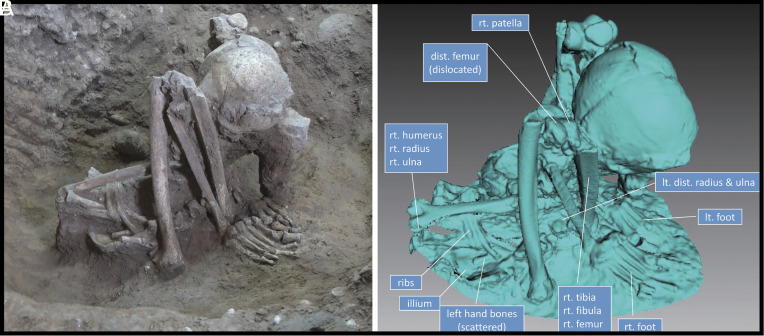
M26, a tightly flexed human burial from Huiyaotian, Guangxi, southern China. (*A*) Skeletal position of a middle-aged male, and (*B*) surface scan of Burial M26. The mostly complete skeleton retains its original articulated position. The pelvis lies on the ground, while the *Right* and *Left* femora are nearly vertical, with their distal ends oriented upward. Both knees are strongly flexed, positioning the feet just medial to the hip joints, and the foot bones are largely intact. The shoulders, rib cage, and upper limbs have collapsed downward, but the positions of the humerus, radius, and ulna indicate that the arms were crossed beneath the thighs, with each hand placed under the opposite knee. Overall, the body is folded in an extremely compact manner (28).

Among the many questions encountered in interpreting these burial practices, one major puzzle relates to the hyper-flexed postures of some individuals, postures that appear impossible to achieve without extraordinary intervention prior to interment. For instance, Huiyaotian M26 (Fig. 4) is mostly complete and retains its original articulation, but it is folded in a very compact manner (28). How was this degree of contortion achieved?

In primary burials encased in a sedimentary deposit, the decomposition process will lead to the creation of empty spaces as the soft tissue disappears. Disarticulated small bones, such as phalanges, will often move within these voids. However, our observations at several sites in northern Vietnam and southern China, including Huiyaotian and Liyupo, reveal that many of the hyper-flexed burials were so tightly contorted that no empty spaces existed between limbs and torsos. This suggests that no soft tissues, apart perhaps from dried skin, remained around the skeletons when they were buried.

Similar findings related to this phenomenon have been discussed in other parts of the world. As an example, a recent study of European Mesolithic burials from the Sado Valley in Portugal reports that the feet and lower limbs of some individuals exhibit extreme flexion alongside unexpectedly well-preserved labile articulations. The authors suggest that the bodies were not buried as fresh cadavers but rather in a desiccated state, indicative of some kind of deliberate post-mortem treatment (12).

**Fig. 5.**
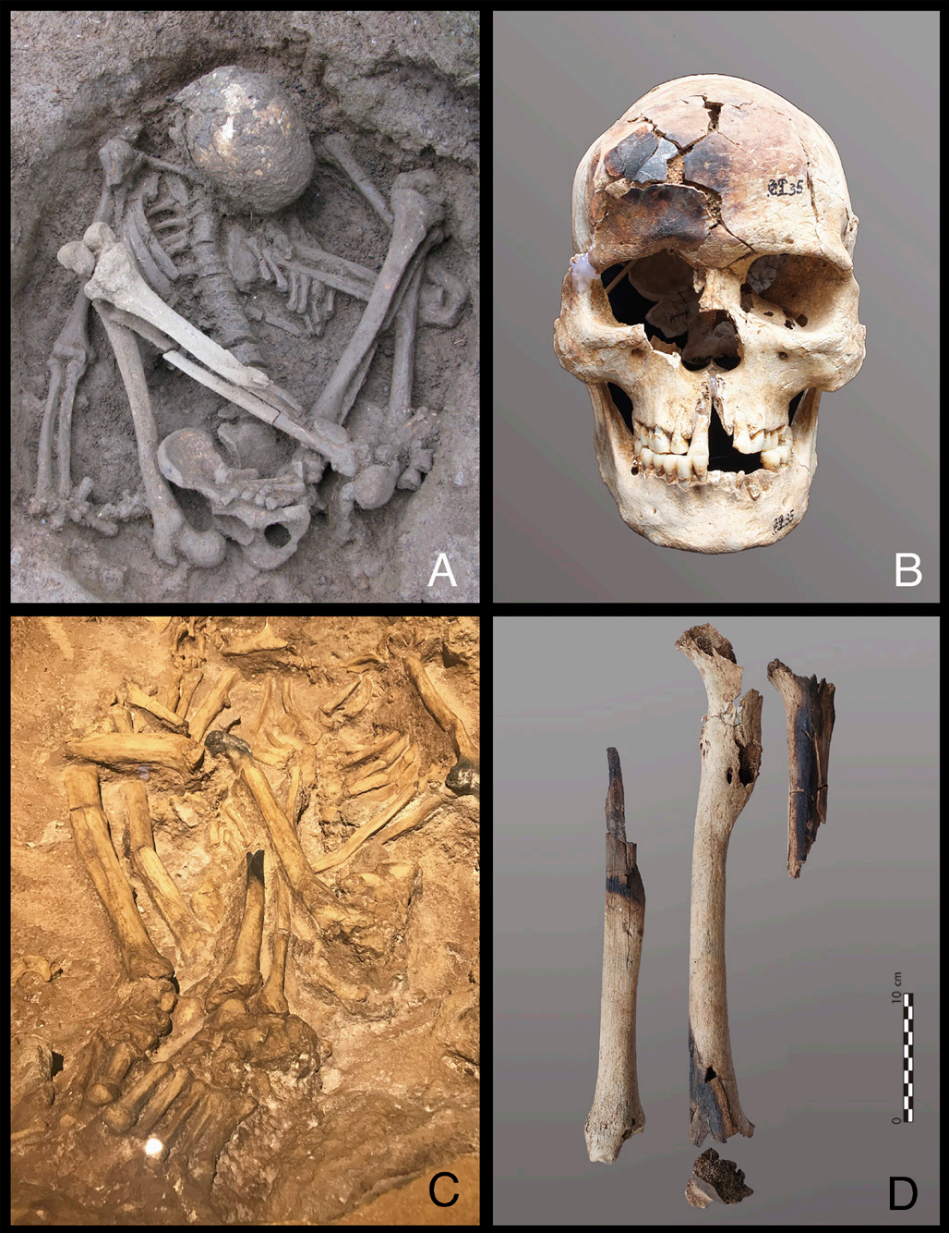
Examples of hyper-flexed burials with partially burned bones from southern China and Indonesia. (*A* and *B*) Burial M35, a young male from Liyupo in Guangxi, with its partially burned skull; (*C* and *D*) Burial ST1 from Song Terus Cave in Java, showing its partially burned left femur, tibia, and humerus (*C*: replica; *D*: courtesy Indonesian-French Joint Prehistory Program).

Another question addressed to our burial sample is whether or not there is evidence for a use of fire and smoke in the treatment of the corpses before burial. At Huiyaotian and Liyupo in Guangxi, some bones exhibit clear signs of exposure to fire. This is indicated by their burnt and blackened conditions, even though they might still be in articulation, as in a primary burial. The majority of such burned bones include the frontal bones of the skull (Fig. 5 *A* and *B*), the lower limb bones such as the femur, tibia, and fibula, the upper limb bones in the elbow region including the humerus, ulna, and radius, and the pelvic bones (*SI Appendix,* Table S2). Bones with traces of burning were identified in approximately 15% of the 75 individuals sampled from Huiyaotian and Liyupo.

Similar examples of flexed, contorted, and sometimes partially burnt burials have also been excavated from sites in northern Vietnam, Sarawak, southern Java, and northern Palawan (Philippines) (*SI Appendix,* Table S1). An intensely burned proximal end of the right ulna was recovered from Burial M1 at Hang Muoi Cave (63HMM1) in Hoa Binh Province, northern Vietnam. The remains are those of an adult male exhibiting pronounced cranial features characteristic of Indigenous Australo-Papuan populations (29, 30). A charred human clavicle fragment from the same Hang Muoi burial context yielded a radiocarbon date of 14,027–13,798 cal. BP (HANGHUM19660) (30). Not far from Hang Muoi, a partially burned cranium (87DCM1), attributed to an older adult male of a similar chronological period, was excavated from the Dong Can site (29).

Tom and Barbara Harrisson recorded burned burials from the West Mouth of the Niah Caves in Sarawak during their excavations in the 1950s and 1960s. Among the more than 150 burials they recovered, 39 were classified as Mesolithic or pre-Neolithic, including examples of flexed, seated, and mutilated burials (31). The estimated temporal range of these Mesolithic ritual practices spans from 11,698–11,270 cal. BP (OxA-15157) to 8,454–8,354 cal. BP (OxA-16161) (32). Three burials with seated postures (B54, B141, and B147) were thought to have been placed on fires. Burial 147 was the best-preserved, and, according to the field notes kept by Barbara Harrisson,

“…lifting the hip bones we find that charring is heavier on the underside….there is no black on the top of the skull, but the teeth and lower jaw under [the] skull below is black. The teeth...even seem burned” (33). Lindsay Lloyd-Smith, who excavated more recently at Niah, further noted that “…for whatever reason (functional, symbolic or aesthetic) the sitting position was desired for those burials which were placed in a fire pit”(34).

The Song Terus specimen (ST1) from southern Java, directly dated to 8,500 cal. BP (35), was buried in an articulated and flexed posture, also with prominent charring on the left side of the body, including on the left femur, tibia, and humerus (Fig. 5 *C* and *D*) (36). Another Early Holocene burial in Ille Cave, northern Palawan, contained compact bone fragments from a single human skeleton (C758), directly dated to 9,006–9,260 cal. BP (OxA-16020) and 9,280–9,425 cal. BP (OxA-15982), with evidence of burning and cut marks indicative of disarticulation (37).

Given the predominance of evidence for burning in some anatomical areas, but not across the whole skeleton, the intended treatment most likely was not full cremation. Instead, the charring appears localized and consistently associated with specific anatomical areas, particularly on the lower limbs, elbows, and frontal areas of the cranium. This pattern suggests that the bodies may have been arranged in tightly flexed or seated positions and exposed to heat in a controlled or semi-controlled environment prior to burial. Most plausibly, the remains underwent a treatment involving proximity to fire, such as smoking, that affected particular skeletal areas more prone to burning, especially in areas with less muscle mass and thinner soft tissue coverage. If this interpretation is correct, then even skeletal elements that lack visible signs of burning may preserve subtle structural or compositional changes indicative of heat exposure, potentially testable through further analysis.

## Results

### XRD Analysis.

To assess potential heat exposure in the analyzed skeletal remains, XRD analysis was conducted on 20 archaeological bone samples from sites in Vietnam, alongside two sub-modern control samples from Edo Castle in Tokyo (*SI Appendix*, Table S2, Sample #70 and #71). As attested by previous research, XRD is particularly effective in detecting high-temperature thermal alterations, as heating above 500 °C leads to significant hydroxyapatite recrystallization, resulting in sharpened diffraction peaks and structural reorganization. The diffraction patterns obtained were compared to reference datasets to classify the samples according to their thermal exposure (*SI Appendix*, Table S3 and Figs. S2 and S3-1 and S3-2). Based on the comparisons with Shipman’s temperature-based criteria (38), the results were as follows:

1. Heated Samples (> 525 °C, or >645 °C): Nine target samples and one heated control sample exhibited highly sharpened peaks in the 32 to 34° 2θ range, indicating heating above 525 °C, with some showing characteristics of exposure to temperatures exceeding 645 °C. These samples are classified (*SI Appendix*, Table S3 and Figs. S3-1 *A*, *C*, *D*–*F*, *I* and *L* and S3-2 *O* and *R*) as >525 °C or >645 °C, confirming significant thermal alteration.
2. Likely Unheated Samples: Three samples displayed diffraction patterns with no significant crystallographic changes indicative of burning, similar to the unheated control sample. These are labeled “unheated” (*SI Appendix*, Table S3 and Figs. S3-1 *G* and *J* and S3-2 *Q*), suggesting no exposure to high temperatures.
3. Probably Heated Samples (~525 °C): The remaining eight samples exhibited patterns resembling those of bones heated to approximately 525 °C. However, similar patterns can also result from long-term diagenetic processes. These samples are therefore labeled ~525 °C (*SI Appendix*, Table S3 and Figs. S3-1 *B*, *H*, and *K* and S3-2 *M*, *N*, *P*, *S*, and *T*), indicating that while they were likely exposed to heat, diagenetic influences cannot be ruled out. Nevertheless, the three unheated samples mentioned under 2 above, which came from the same geological environment as the eight probably heated samples, did not show hydroxyapatite growth. This suggests that the latter were possibly heated at low temperatures.

### FTIR Analysis.

While XRD effectively identifies high-temperature thermal alteration, it is less sensitive to low-temperature heating, which does not always induce detectable changes in hydroxyapatite crystallinity. Additionally, some samples displaying moderate crystallographic modifications may have undergone diagenetic alteration rather than heat exposure. To address these limitations and further investigate heat treatment, particularly in cases without visible burn marks, we employed FTIR on 69 bone samples from 11 pre-Neolithic sites in southern China, northern Vietnam, and Indonesia. One sub-modern control from Edo Castle (intentionally burned) was included for reference (*SI Appendix*, Tables S2 and S4).

**Fig. 6.**
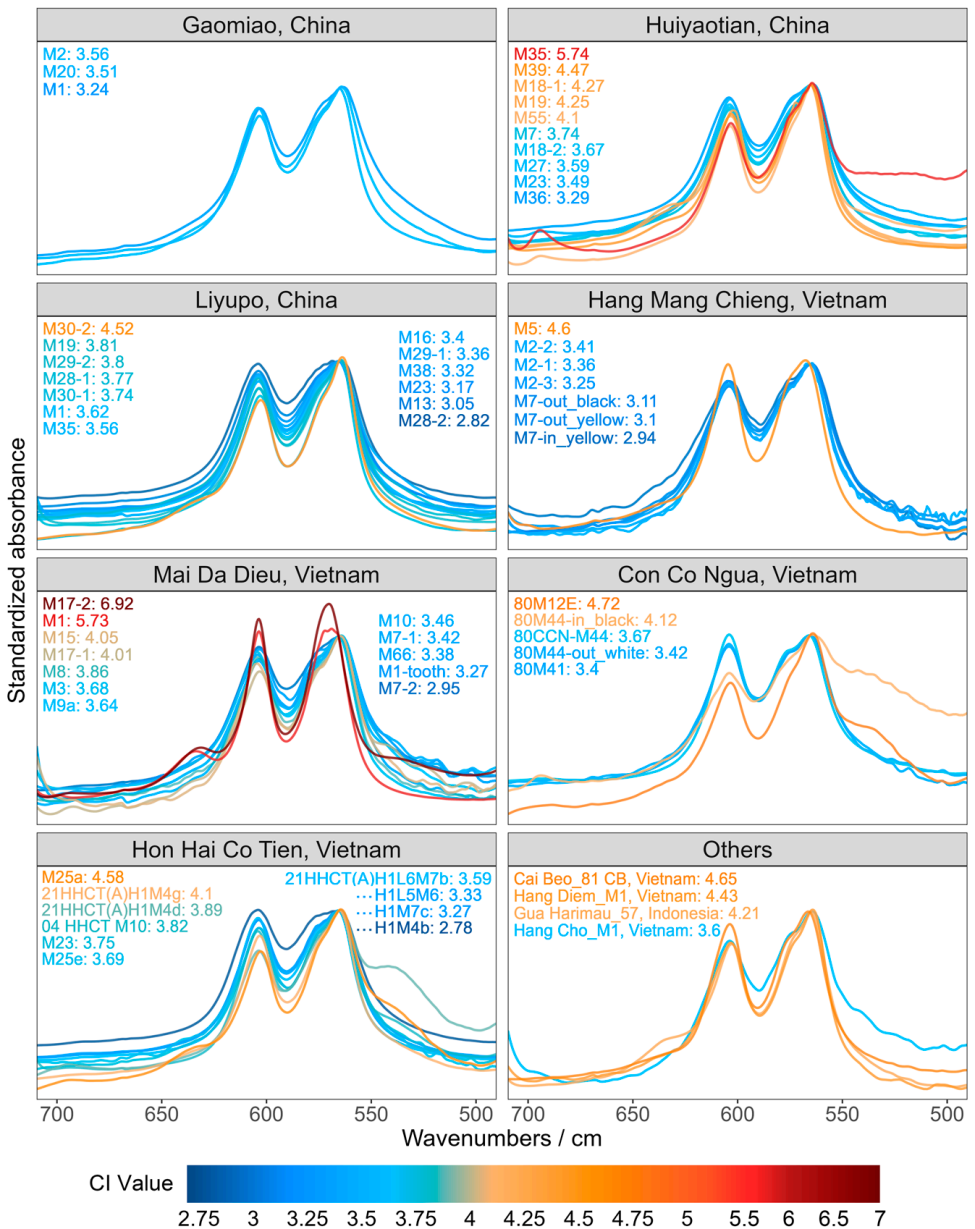
Standardized FTIR absorption spectra (710 to 490 cm^−1^) of human bone samples, color-coded by CI values. Tested samples include those from Gaomiao, Huiyaotian, and Liyupo in China; Hang Mang Chieng, Mai Da Dieu, Con Co Ngua, and Hon Hai Co Tien in Vietnam; as well as additional samples from Cai Beo, Hang Diem, and Hang Cho (Vietnam), and Gua Harimau (Indonesia).

Among the 69 analyzed target samples, 64 produced reliable FTIR data, while five exhibited poor signal quality and were excluded from further analysis (Fig. 6 and *SI Appendix,* Table S4). The Crystallinity Index (CI) of each sample was then calculated to assess the degree of heat exposure. As demonstrated by previous research, CI values increase with heating or diagenetic alteration, with modern, unaltered bones typically having CI values between 2.5 and 3.25. CI values above 3.25 suggest possible heat exposure, while those exceeding 3.8 indicate high temperature burning (39–41). One control sample, an intentionally burned human bone from Edo Castle (*SI Appendix*, Sample #71), aligned well with these criteria since it had a CI value of 4.42.

Regarding the 64 targeted samples, the FTIR results indicate that heat exposure was quite common, with a total of 84.38% of samples having CI values above 3.25. Eight samples had CI values below 3.25, indicating no evidence of heat exposure. 33 fell between 3.25 and 3.80, suggesting low temperature heating with diagenetic alteration. 23 samples had CI values that exceeded 3.80, providing evidence for relatively high temperature burning.

Further analysis of parallel samples from the same individuals revealed significant variation in CI values between different skeletal elements. For instance, in burial M18 from Huiyaotian, the left proximal ulna had a CI value of 4.27 (*SI Appendix*, Sample #6), whereas the midshaft of the same bone measured only 3.67 (*SI Appendix*, Sample #7). Similarly, in burial M1 from Mai Da Dieu, the talus had a CI value of 5.73 (*SI Appendix*, Sample #41), while a tooth from the same individual yielded a much lower value of 3.68 (*SI Appendix*, Sample #43). This intra-skeletal heterogeneity in CI values within individual skeletons rules out the possibility of uniform diagenetic alteration resulting from burial conditions alone. Instead, this patterned divergence reflects different degrees of thermal exposure. Consistent with the distribution of macroscopic burn marks, regions characterized by reduced soft tissue coverage and structurally vulnerable epiphyseal zones, such as the front of the skull, the distal end of the femur, and the proximal end of the tibia, demonstrate preferential thermal alteration on both macroscopic and microscopic scales.

While thermal modification in the analyzed sample appears widespread, the majority of the tested bones reflected only low intensity heating. CI values clustered between 2.78 and 4.72, with only three outliers that exceeded this range – one from Huiyaotian (*SI Appendix*, Sample #12) and two from Mai Da Dieu (*SI Appendix*, Samples #41 and #51) (Fig. 6 and *SI Appendix*, Table S4). These results indicate only sporadic and localized high temperature exposure. This aligns with the scarcity of macroscopic burn marks and the near-absence of the peak corresponding to the OH^−^ libration mode at approximately 630 cm^−1^, a spectral signature diagnostic of heating above 400 °C. Among the 64 samples tested, this peak was detectable in only 11 cases and prominent in only four, further corroborating the dominance of low temperature thermal regimes.

Additional insights emerge from the analysis of the blackened bones. In conventional cremation contexts, charred bone (as opposed to ash) results from direct flame contact at moderate temperatures (42). Such cases are identifiable via FTIR, as exemplified by the humerus fragment from Con Co Ngua M44 (Fig. 6). The blackened inner portion of this bone yielded a CI of 4.12 (*SI Appendix*, Sample #57), contrasting with the surface of the bone which had a CI of only 3.42 (*SI Appendix*, Sample #58). This disparity reflects the trabecular bone’s heightened susceptibility to thermal degradation due to its porous architecture. However, such charred specimens are rare in our dataset.

From our FTIR results we infer a mechanism that did not involve intentional direct burning to explain part of the observed discolorations, this mechanism being smoke-induced blackening. The fibula from Hang Mang Chieng burial M7 (*SI Appendix*, Samples #65–67) exemplifies this phenomenon. Its surface displays alternating blackened and yellowish patches, while the interior remains uniformly yellowish (Fig. 6). Sub-sampling revealed nearly identical CI values for both surface zones (3.11 vs. 3.10) (*SI Appendix*, Samples #66 and #67), with the interior slightly lower (2.94) (*SI Appendix*, Sample #65). This uniformity, coupled with the absence of elevated CI values, confirms that the observed discoloration arose from soot deposition in a low-oxygen, smoky environment, rather than from direct combustion.

**Fig. 7.**
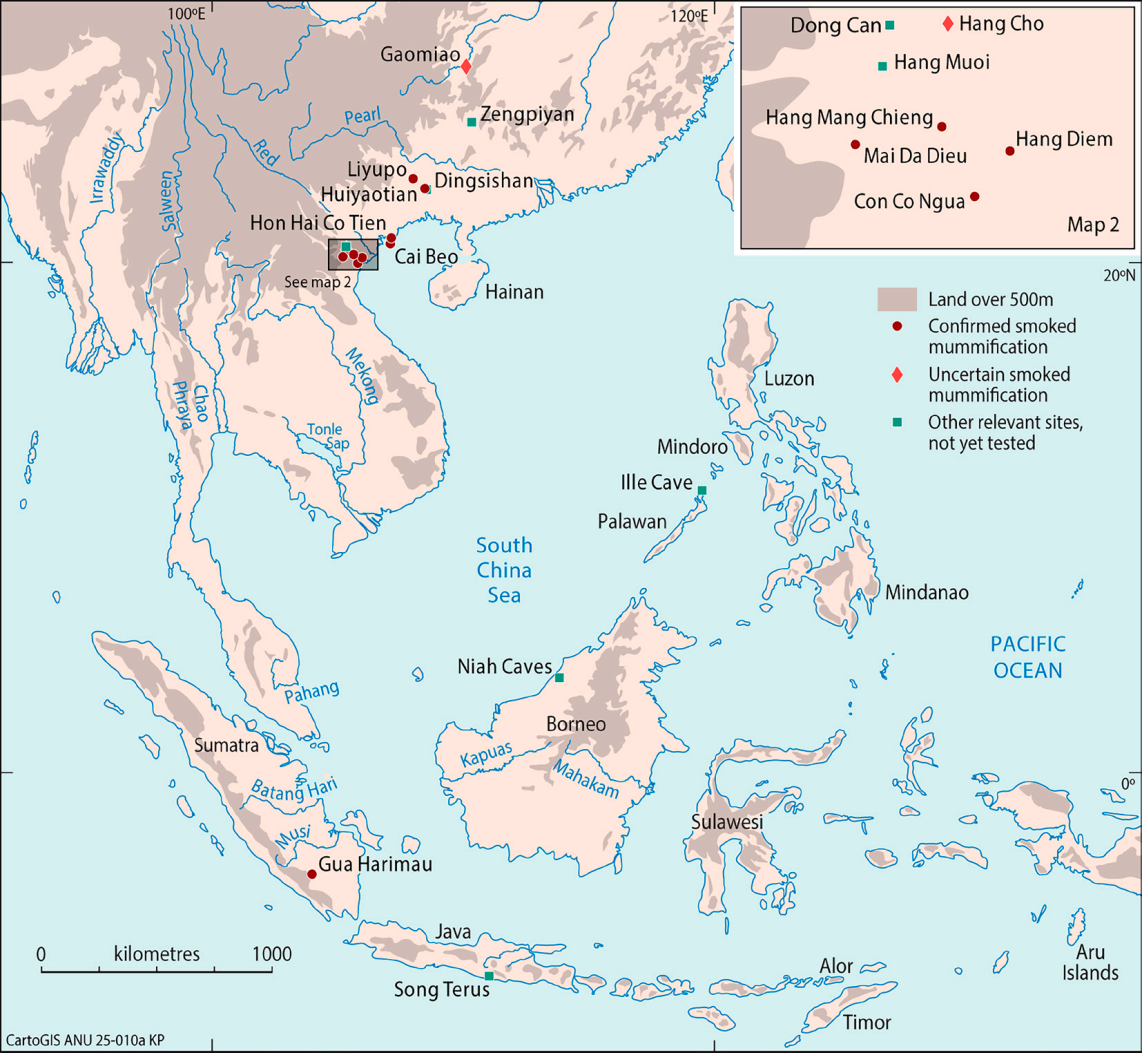
Locations of nine sites with confirmed mummification by smoking identified in this study, along with two uncertain and four relevant sites mentioned in the text. This study analyzed burial samples from 11 sites, and confirmed that smoke-dried mummification occurred at nine of them: Huiyaotian, Liyupo, Hon Hai Co Tien, Cai Beo, Hang Mang Chieng, Mai Da Dieu, Hang Diem, Con Co Ngua, and Gua Harimau. Two other sites, Gaomiao and Hang Cho, remain uncertain due to their weaker FTIR signals. Beyond the 11 primary study sites in China and Vietnam, the six additional sites of Zengpiyan, Hang Muoi, Dong Can, Ille Cave, Niah West Mouth, and Song Terus, although not yet tested by XRD and FTIR, present strong evidence of comparable cultural practices, as discussed in the main text.

Taken together, these XRD and FTIR results suggest that the examined individuals underwent a distinct form of heat treatment before burial. Different body parts were exposed to relatively low temperatures, with only certain skeletal regions being subjected to direct burning. Some blackened areas may have resulted from incidental burning, while others were likely caused by prolonged exposure to smoke rather than open flames. This pattern of heat exposure suggests a specialized mortuary practice in the pre-farming communities that existed across southern China and Southeast Asia (Fig. 7). Unlike conventional cremation, which involves uniform burning of the entire body, this treatment appears to have involved controlled heat application, or smoke exposure.

## Discussion

Ancient human societies are often best known from their burial traditions, especially those that used methods of corpse preparation to enhance the preservation of ancestors, literally in the flesh. A well-known early tradition of mummification involving desiccation is reported from the Chinchorro culture along the arid Atacama coastline of western South America from around 7,000 cal. BP, although the practice apparently was abandoned around 3,700 cal. BP (43). The earliest Chinchorro mummies are those of children whose bodies were desiccated in the arid climatic conditions after their viscera and brains were removed and their legs, arms, and trunks reinforced with sticks. Their faces were coated with a thin layer of clay and painted. Several different Chinchorro mummification styles are recognized by archaeologists working in this region (43, 44).

Ancient Egyptian mummies are deservedly the most famous and extensively studied of the world’s desiccated and embalmed ancient burials, not only because of their cultural and artistic accoutrements, such as painted sarcophagi and valuable grave goods, but also because of the vast quantity of textual material that allows interpretation of the beliefs that fueled their preparation. Among the ancient Egyptians, a deceased body was preserved with the intention of making it incorruptible and ensuring that it resembled the individual’s living appearance (44, 45). The earliest attempts at mummification with embalming date from the Old Kingdom (ca. 4,500 cal. BP), although older Predynastic desiccated burials are also preserved in the Nile Valley. Over time, generations of practitioners refined the embalming techniques and the related traditions, continuing into the Roman Period with realistic painted sarcophagus portraits of the faces of deceased individuals (46).

The evidence for mummification discussed in this report appears to have commenced before either the Chilean or the Egyptian traditions, and it comes from humid monsoon rainfall regions in East and Southeast Asia. In these climatic conditions, natural desiccation was not possible, and we present evidence to suggest that corpses were instead smoked to cure and mummify the skins around their skeletons. The specimens concerned are from hunter-gatherer archaeological contexts in East and Southeast Asia, all excavated as inhumed and still-articulated skeletons, except when bones were disturbed or deliberately rearranged. They date from the Late Pleistocene to the Middle Holocene and the majority are documented from caves, rock shelters, and shell middens. Associated cultural assemblages, when recovered, are Epipalaeolithic (usually termed “Hoabinhian” in Mainland Southeast Asia), with pebble, core and flake industries that lack blade and biface elements. Some sites in southern China and northern Vietnam are Para-Neolithic in the sense of Bellwood 2017 (4), with fired earthenware pottery and ground pebble axes. None reveal evidence for food production with domesticated crops or animals, or contain artifact assemblages with stylistic features typical of more recent Neolithic cultures. Unlike Neolithic burials in Southeast Asia, these pre-Neolithic burials were not usually provided with grave goods.

### Similarities Between Ancient Burial Remains and Contemporary Mummification in Papua and Australia.

**Fig. 8.**
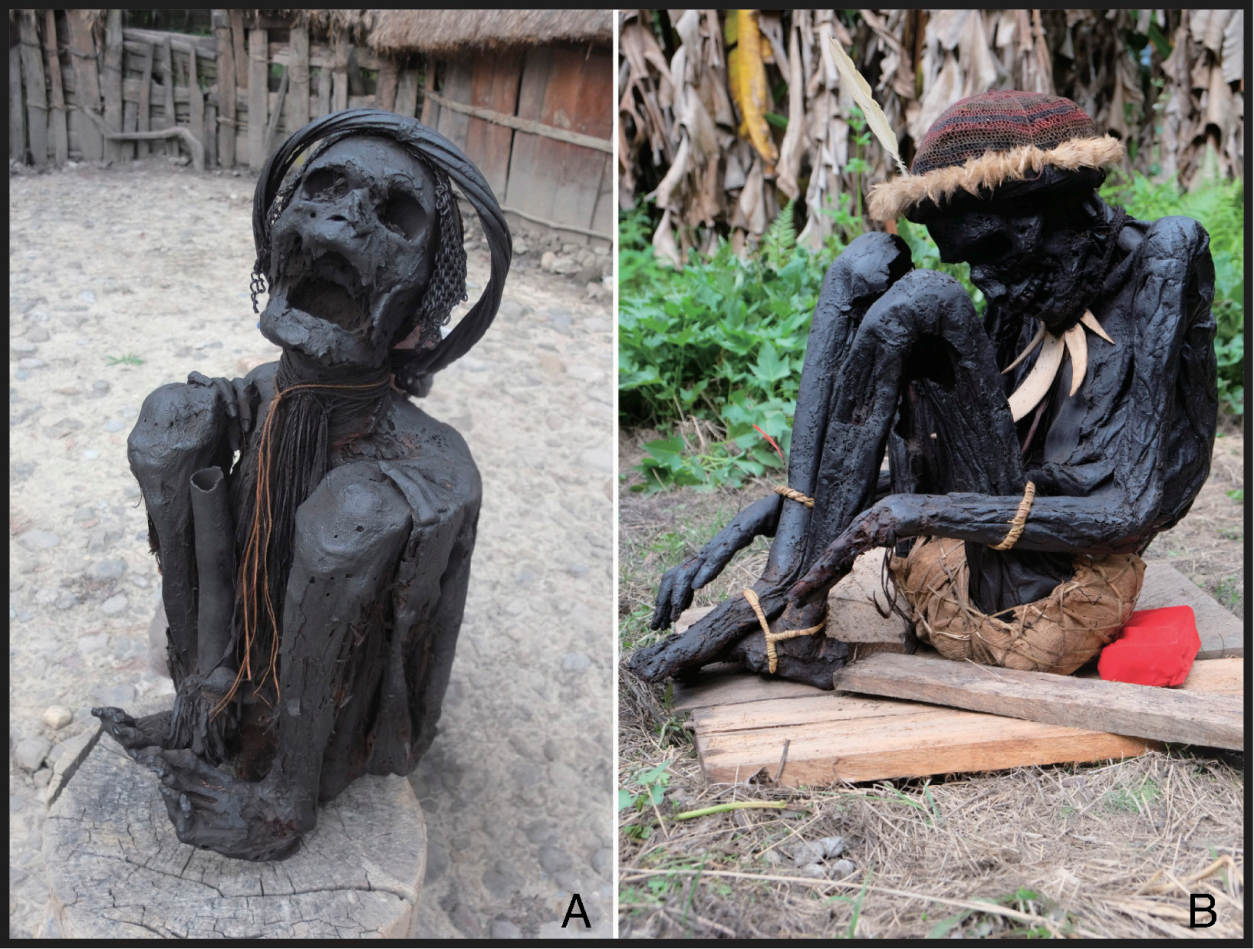
Examples of smoked mummies kept in private households in Papua, Indonesia, photographed in January 2019. (*A*) A Dani hyper-flexed mummy; (*B*) A flexed mummy from Pumo Village. Both locations are in Wamena, Jayawijaya Regency, Papua.

To understand this unique mummification practice better, modern ethnographic materials may provide further comparative insights and references. In January 2019, our team conducted a field survey of ethnographic smoked mummies among the Dani and Pumo people who live 1500 m above sea level in the Baliem Valley, Jayawijaya Regency, Papua (Indonesian New Guinea). Among the Dani, mummification practices involved the smoking of the body until it turned entirely black. The Dani mummies were often tightly compressed with their four limbs bound to the trunk, an action presumably undertaken immediately after death and prior to the onset of rigor mortis, although the actual degrees of flexion varied and some appear to have been unbound (Fig. 8). Among the Dani, the descendants kept the mummy in a special room within the house, and would bring it out on special occasions. These highly compact examples are very similar to those from our study sites in Southeastern Asia (Figs. 2 and 3 and *SI Appendix,* Figs. S1 and S4).

Another example of New Guinea Highland mummification involves the Anga (Kukukuku) people who reside in the Aseki region of Morobe Province in eastern Papua New Guinea (13–15, 47). Traditionally, several Anga groups mummified their revered ancestors, including warriors, shamans, and village leaders, by smoking their bodies. The deceased were placed in a seated position above a continuously burning fire, similar to the practice of the Dani people, and the smoking process typically lasted for around three months (14). When it was considered to be sufficiently mummified, the resulting corpse was displayed on a protected ledge or niche in a cliff, although in the Anga case, and also among the Dani, there appears to be no evidence for ultimate burial of an articulated skeleton, unlike the archaeological samples under discussion in this paper.

**Fig. 9.**
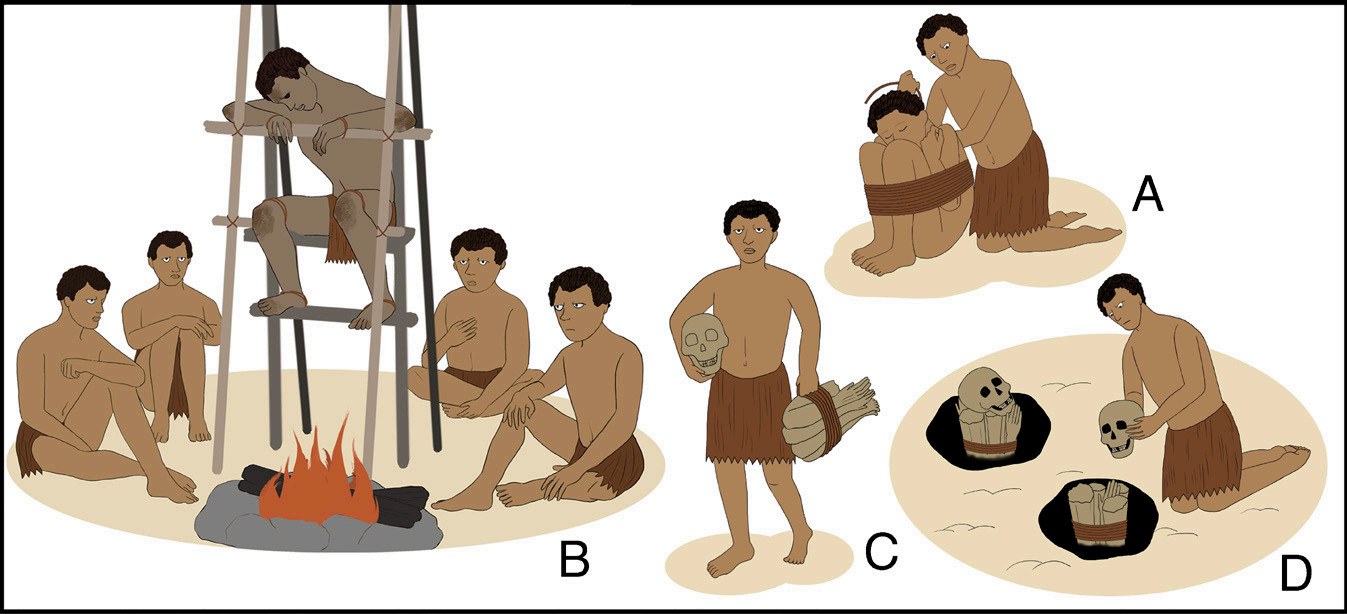
This study proposes a scenario for prehistoric smoke-drying mummification, drawing on both archaeological findings and ethnographic parallels from Papua. The illustration shows the multi-step process of smoke-drying mummification, as ascertained through our study. (*A*) Preparation for the smoking ritual—corpses were bound to varying degrees, often in a hyper-flexed position; (*B*) Processing – the corpse could be bound (as shown in *A*) or unbound (as shown in *B*, based on a modern example in Papua New Guinea, see ref. 14), while placed above a low-temperature fire. In our Late Pleistocene–Middle Holocene examples, all individuals were bound; (*C*) Post-smoking treatment – after the smoking was completed, then the smoked mummy was transferred to a residence, a specially constructed hut, a rock shelter, or a cave. In some cases, however, the mummy decayed due to prolonged exposure to open air. As seen in the example presented here, the head and body became separated; (*D*) Final burial – the smoked mummy eventually was buried (see field photo in *SI Appendix*, Fig. S11). Most were intact at the time of burial. Some cases had decayed by this time in the process, and those remains were rearranged in the burial pit, sometimes appearing as suspected intentionally dismembered burials in the archaeological record.

Drawing upon our observations of ethnographic mummification in the New Guinea Highlands, we suggest that many (but not all) of the ancient deceased individuals were tightly bound immediately after death and suspended above low temperatures and smoky fires for long periods (Fig. 9). The partially blackened and burned bones, as observed at archaeological sites (Fig. 5), may have resulted from fires that occasionally became uncontrolled during the smoking process, possibly when additional fuel was added. Although people may conceivably have lit fires directly in graves, for instance as suggested in the Niah West Mouth (34), we have not identified any such fireplaces located directly within graves in our analyses of excavation records. Ethnographic parallels suggest that the smoking was undertaken somewhere under a roof, perhaps inside a house or a specially constructed hut (14, 48). Once the smoking was complete, the mummy was presumably transferred to a protected residence, rock shelter, or cave for as long as the individual remained within living memory, eventually perhaps to be buried, as were all the skeletal specimens investigated in this study.

**Fig. 10.**
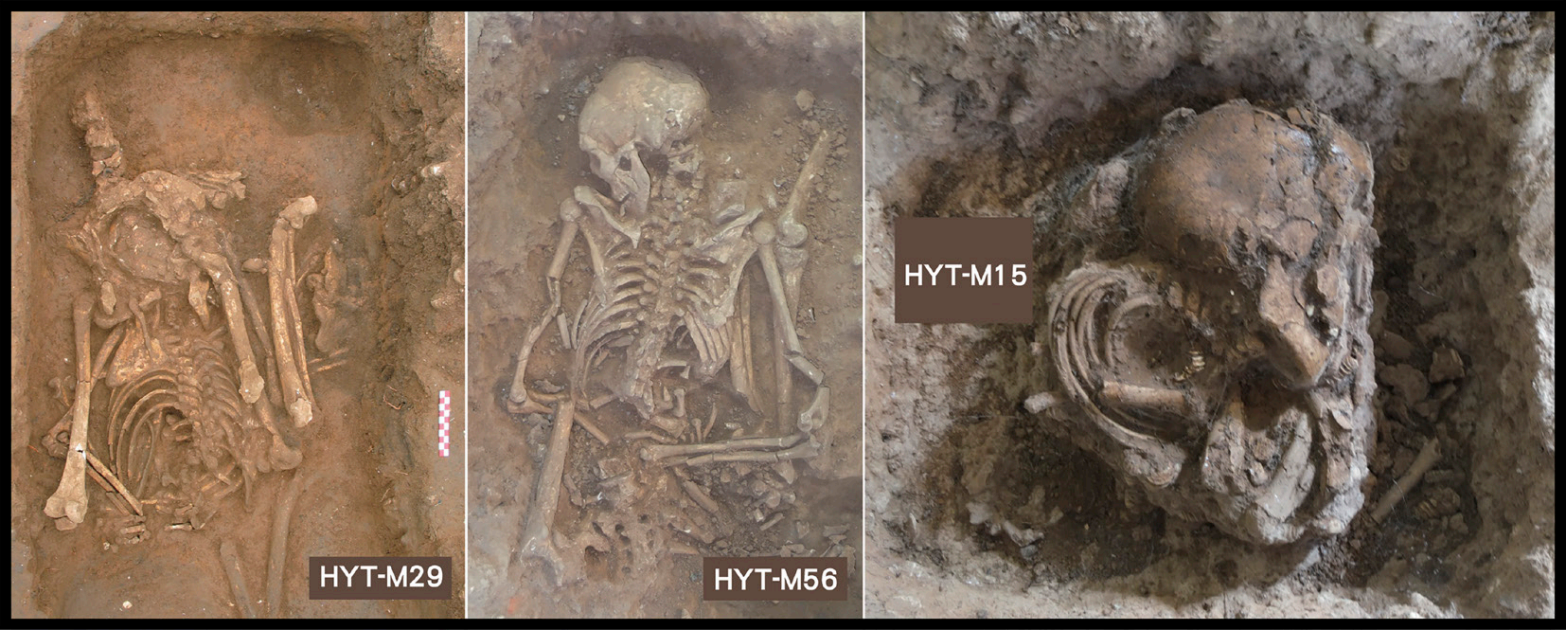
Examples of burials from Huiyaotian (HYT), Guangxi in southern China, previously suspected to be dismembered. HYT-M29 (adult female): the hip and knee are positioned adjacent to each other, which is an anatomically impossible orientation. HYT-M56 (adult male): the backbone lies face upwards, but the hip lies prone, again an anatomically impossible orientation. HYT-M15 (child aged 3 to 5 y): highly compacted and bundled bones.

Ethnographic analogies clarify some aspects about the disarticulated or misaligned bones as well as about the cut marks observed on some of the burials. At sites such as Dingsishan (49), Huiyaotian, and Liyupo in Guangxi, certain individuals were found with anatomical anomalies, for example, skulls placed within thoracic cavities, or lower legs arranged where forearms would normally be located (*SI Appendix,* Fig. S5). At Huiyaotian, one individual exhibited a complete torsional displacement of the trunk over the lumbar region, while others included upright bundles of long bones resembling tied firewood (Fig. 10). Such configurations were previously interpreted as evidence of intentional dismemberment (50, 51), possibly associated with mortuary rituals. Similar findings have been reported at other sites in Southeast Asia, such as Niah, where they were described as “mutilation burials” (31).

Given the ethnographic findings, we suggest that the disarticulated bones within ancient skeletal remains reflect post-mummification processes. Exposure over extended periods, decay of soft tissues, or disturbance during transport may have led to joint loosening and partial disarticulation. Misalignment or unconventional positioning of bones could have occurred during burial, especially if people attempted to reassemble or reposition the remains after decay had begun. This perspective reframes such features as taphonomic outcomes of the smoked mummification process rather than as indicators of ritual dismemberment (*SI Appendix*, *Text*).

The confirmation of smoked mummification among these ancient societies allows us to re-examine the presence of cut marks on some of those skeletons, which have been reported at many sites in Southeastern Asia (e.g., 36, 37, 50–52). At sites in Guangxi, they had previously been interpreted as evidence of intentional dismemberment. At Huiyaotian, cut marks were observed on 19 out of 100 examined individuals, concentrated primarily around the epiphyses of major limb bones such as the humerus and femur (52) (*SI Appendix*, *Text* and Figs. S6 and S7 and Table S5). These marks can be understood as anatomical interventions designed to facilitate joint release or enable tighter postmortem flexion, particularly if binding was delayed and rigor mortis had already set in. In some cases, cuts may have served to allow drainage of bodily fluids during smoking.

A smaller subset of marks, found notably on the femora and tibiae, are consistent with post-smoking defleshing (*SI Appendix*, Figs. S6 and S8), possibly reflecting ritual acts after the body had been mummified. An ethnographic account of the creation of tightly bound smoked burials in Aboriginal South Australia describes instances wherein relatives removed small pieces of flesh from the thigh of a corpse to offer to a sorcerer in exchange for magical protection or retribution (48). Such practices may offer insights into similar defleshing marks observed on burials at Huiyaotian and several other sites, such as Ille Cave in Palawan, where both cut marks and “scrape marks” are documented (37).

The ensemble of skeletal and taphonomic evidence points to a complex mortuary tradition involving smoked mummification, delayed burial, and, in some cases, ritual interaction with the post-mummification body. These patterns represent a coherent and intentional framework for treating the dead, embedded in belief systems that possibly date back at least 10,000 y.

One might ask why people practiced this kind of mummification across a broad area that might once have encompassed much, if not all, of Southeastern Asia, New Guinea, and Australia. In practical terms, smoking was likely the most effective option for preserving corpses in tropical climates, where heat and humidity would otherwise have caused rapid decomposition. Nonetheless, the consistency and care in these treatments may suggest that preservation alone was not the sole consideration. Among the Anga, for instance, people still believe that the spirit of the deceased roams freely during the day and returns to the mummified body at night (14). Among the Taramindjeri of South Australia, mummification is linked to a hope of immortality (48). These beliefs highlight the types of symbolism that might have been attached to the body and its treatment after death.

### Millennia of Smoked Mummification in Early Hunter-Gatherer Societies.

One important question still remains. Do the similarities between the smoke-mummification techniques recorded from southern China to New Guinea and southern Australia, from contexts that date from the Late Pleistocene into ethnographic times, reflect a shared cultural tradition, one that could potentially go back as far as the early expansion of *Homo sapiens* from Africa through tropical latitudes of southern Asia? Or are the similarities purely coincidental?

Recently analyzed genomic, craniofacial, and archaeological evidence suggests that pre-Neolithic Southeastern Asian populations, along with the ancestors of modern Indigenous Papuans and Australians, were closely related genetically and phenotypically to the early *Homo sapiens* who migrated out of Africa through tropical Asia. These ancient hunter-gatherers were distinct from the later Neolithic farming populations whose migrations originated farther north during the Holocene, such as those associated with Austroasiatic, Kra-Dai, and Austronesian ancestry (1–6, 53–55). Craniofacial studies by Hirofumi Matsumura and colleagues support a two-layer model of Southeastern Asian prehistory, identifying an early “first layer” population ancestral to modern Negrito groups (Andaman Islands, Peninsular Malaysia, Philippines), Indigenous Australians, New Guinea Papuans, and ancient Jomon people of Japan (5, 53) (*SI Appendix,* Figs. S9 and S10). The mummified individuals analyzed in this study belonged in terms of their craniofacial morphology to this initial layer of anatomically modern human population in Southeastern Asia (5).

This perspective suggests that smoked mummification might have originated earlier, and been more widespread, than is currently identified in the archaeological record. Flexed burials, often associated with partial burning or hyperflexion, have been documented at multiple sites across Southeastern Asia in contexts likely older than 12,000 cal. BP. Relevant sites include Dayan (15,000–12,000 cal. BP), Miaoyan (19,000–12,000 cal. BP), Fengyan (42,000–33,000 cal. BP), Zhaoguodong (12,000–9,000 cal. BP), Huangmenyan (14,000–10,000 cal. BP) (56–58), and Hang Muoi Cave (with a direct C14 date on a human clavicle of 14,027–13,798 cal. BP; HANGHUM19660) (29, 30).

Similar mortuary practices may have been present in Northeast Asia, particularly during the Initial Jomon period (11,500–7,000 cal. BP) in Japan (8), and in Korea during the pre-farming period on Gadokto Island (6,600–6,300 cal. BP), southeast of the Korean Peninsula (9, 59), where contorted burial postures have also been recorded. Furthermore, the Sakhalin Ainu practiced sun-drying mummification well into the early 19th century (60). A few sites in northern China that have flexed burials (7, 61) dated between 13,000 and 10,000 cal. BP would also benefit from further investigation.

Further parallels can be found in the tightly flexed and hyper-flexed burials recovered in several sites in Australia (11, 62, 63). The Broadbeach burial ground in Queensland was excavated in the 1960s (62), and its burials show striking similarities in their bundled postures to the burials examined in this study. While the Broadbeach burials were not directly dated by C14, the excavator believed them to be late prehistoric and the descriptions of dismemberment and binding align closely with the practices observed in our study.

Our findings highlight a deep and enduring biological and cultural continuity, linking ancient hunter-gatherer populations in Southeastern Asia with modern Indigenous communities in New Guinea and Australia. The tradition of smoked mummification serves as compelling evidence of long-term cultural persistence between ancient Southeastern Asian and ethnographic Papuan and Australian mortuary practices. Furthermore, archaeological findings suggest that this tradition may have been known among hunter-gatherer societies across a vast region, for many millennia, extending from northeastern Asia and Jomon Japan to western Oceania and Australia, and possibly farther. Through this practice, the smoked and preserved remains of the deceased allowed people to sustain physical and spiritual connections with their ancestors, bridging time and memory.

## Materials and Methods

### Provenance and Examination of Human Remains.

The human remains analyzed in this study were collected from archaeological sites through multiple research projects conducted across different countries. Their authenticity, contextual integrity, and estimated ages have been validated by scholars through previously published studies. *SI Appendix*, Tables S1 and S2 provide detailed information for each site, including its location and relevant academic references. The timeline and personnel involved in the recovery of the remains are documented in the corresponding publications. This study offers additional direct radiocarbon dating of some of the human skeletal remains (*SI Appendix,* Table S2).

Between 2017 and 2025, our team examined these remains at their respective host institutions (see *SI Appendix,* Table S2 for institutional details). Initial analyses included both on-site and laboratory-based physical anthropological investigations, involving macroscopic examination and forensic assessment of the skeletal remains, with particular attention to anatomical positioning and key morphological features. Burn patterns, whether visibly present or absent on the remains, were correlated with scientific analyses to evaluate the extent and nature of thermal exposure.

Small portions of selected samples from each site were obtained for laboratory analysis through official procedures, with permits granted by the respective institutions. The majority of the human remains continue to be curated at their original institutions. Future research may be conducted through formal applications for access, in accordance with the policies of the institutions housing the collections.

### XRD Analysis.

XRD analysis was conducted on 20 bone samples (*SI Appendix,* Tables S2 and S3), which were from the same individuals as used in the FTIR analysis. These samples were not heavily carbonized or visibly burned, although they exhibited a relatively bright, whitish color.

The bone fragments were ground into fine powder using an agate mortar and pestle before analysis. XRD analysis was performed using a RINT2000 series X-ray diffractometer (Rigaku Co.). The X-ray beam was generated at 40 kV and 100 mA using a rotating Cu target. The scan parameters were set with a sampling width of 0.02, an operational range of 20 to 45° 2θ, and a scan speed of 2°/min.

The XRD patterns of the samples were compared with diagnostic reference data from Shipman et al., who tested bone samples heated at various controlled temperatures in a muffle furnace (185 °C to 940 °C). Their study confirmed the presence of hydroxyapatite in bones across all tested temperatures (38). Bone samples heated above 645 °C undergo instantaneous diagenesis, whereas those heated below this temperature experience solid-state recrystallization, resulting in clearer and more distinct absorption peaks. This distinction allows differentiation between unheated fossilized bones and bones subjected to low-temperature heating. Furthermore, significant crystal size differences were observed between samples heated above 645 °C and those heated below 525 °C. Heated samples above 645 °C showed peak sharpening and slight narrowing, particularly in the 32 to 34° 2θ range, along with the appearance of narrow peaks in the 26 to 28° 2θ range due to increased peak height. Additionally, reference was made to XRD patterns of control samples: one unheated bone (*SI Appendix*, Sample #70) and one intentionally heated bone (*SI Appendix*, Sample #71) from early modern Edo Castle, Tokyo (*SI Appendix*, Tables S2 and S3 and Figs. S2 and S3-1 and S3-2). The heated sample exhibited distinct sharp peaks in the 32 to 34° 2θ range, consistent with patterns observed for bones heated above 645 °C in Shipman’s study.

### FTIR.

69 bone samples from 57 individuals excavated from 11 hunter-gatherer sites in southern China, Vietnam, and Indonesia were analyzed using FTIR, and some were also subjected to XRD analysis. For 11 individuals, two or three samples were taken from different skeletal regions to assess potential variations in heating intensity across the body. Additionally, these multiple samples allowed for an evaluation of possible preservation-related influences on the FTIR results. Additionally, one deliberately burnt control sample of sub-modern date from Edo Castle (Tokyo) was included to test the reliability of the FTIR analysis. All the tested archaeological samples are curated in local museums or research institutions in their respective countries of excavation (*SI Appendix*, Tables S2 and S4).

FTIR analysis was conducted using a Thermo Fisher Scientific IS5 Fourier Transform Infrared Spectrometer with an iDl transmission accessory. The scanning range was 4,000 to 400 cm^−1^, with 16 scans per sample, a spectral resolution of 4 cm^−1^, and a total collection time of approximately 23 s. Infrared spectra were processed using Thermo Scientific OMNIC software.

For sample preparation, potassium bromide (KBr) powder was dried under an infrared lamp for 40 min and then pressed into a pellet at 4 tons to establish a background spectrum. Approximately 0.1 grams of each bone sample was ground to below 200 mesh, mixed with KBr powder at a 1:150 ratio, and homogenized. The mixture was then pressed into a pellet at 4 tons and analyzed using the FTIR spectrometer.

Following previous research (39–41, 64), the CI value and the presence of a characteristic peak around 630 cm^−1^ were used to determine whether the bones had been subjected to heating. The CI value was calculated as the sum of the peak intensities at 603 cm^−1^ and 565 cm^−1^ divided by the intensity of the trough between them. A secondary indicator of burning is the structural alteration of hydroxyl (OH^−^) groups, which may undergo dehydration or vibrational excitation during combustion. In FTIR spectra, this is manifested as a distinct peak corresponding to the OH^−^ libration mode at approximately 630 cm^−1^ (65). This peak is absent in fresh or archaeological bones that have not been heated beyond 400°. Its presence is considered evidence of exposure to relatively high temperatures.

## Supplementary Material

Appendix 01 (PDF)

## Data Availability

All study data are included in the article and/or *SI Appendix*.
