# Supplementary material for: Earliest evidence of smoke-dried mummification: More than 10,000 years ago in southern China and Southeast Asia
Source: Proc Natl Acad Sci U S A. 2025 Sep 15;122(38):e2515103122. doi: 10.1073/pnas.2515103122 (PMC12478042; doi:10.1073/pnas.2515103122)
Supplement: Supplementary file 1 — Appendix 01 (PDF) [file pnas.2515103122.sapp.pdf]

Supporting Information for

Earliest evidence of smoke-dried mummification: More than 10,000 years ago in southern China and Southeast Asia

Hsiao-chun Hung[a,1,2], Zhenhua Deng[b,c,1,2], Yiheng Liu[b,c], Zhiyu Ran[b,c], Yue Zhang[a], Zhen Li[d], Yousuke Kaifu[e], Qiang Huang[f], Khanh Trung Kien Nguyen[g], Hai Dang Le[h], Guangmao Xie[d,i], Anh Tuan Nguyen[h], Mariko Yamagata[j], Truman Simanjuntak[k], Sofwan Noerwidi[l], Mohammad Ruly Fauzi[l], Marlin Tolla[l], Alpius Wetipo[m], Gang He[n], Junmei Sawada[o], Chi Zhang[c], Peter Bellwood[p], Hirofumi Matsumura[q,1,2]

a. Department of Archaeology and Natural History, School of Culture, History and Language, College of Asia and the Pacific, Australian National University, Canberra, ACT 2601, Australia
b. Key Laboratory of Archaeological Science (Peking University), Ministry of Education, Beijing 100871, China
c. School of Archaeology and Museology, Peking University, Beijing 100871, China
d. Guangxi Institute of Cultural Relic Protection and Archaeology, Nanning 530003, China
e. The University Museum, The University of Tokyo, Tokyo 113-0033, Japan
f. The Dingsishan Site Museum, Nanning 530200, China
g. Center for Archaeology, Southern Institute for Social Sciences, Ho Chi Minh City 700000, Vietnam
h. Vietnam Institute of Archaeology, Academy of Social Science, Hanoi 100000, Vietnam
i. School of History, Culture and Tourism, Guangxi Normal University, Guilin 541006, China
j. College of Arts, Rikkyo University, Tokyo 171-8501, Japan
k. Center for Prehistoric and Austronesian Studies, Jakarta 12710, Indonesia
l. Research Center for Archaeometry, The National Research and Innovation Agency, Jakarta 12710, Indonesia
m. Department of Culture and Tourism, Jayawijaya Regency, Wamena, Papua 99511, Indonesia
n. Institute of Cultural Relics and Archaeology of Hunan, Changsha 410011, China
o. Institute of Physical Anthropology, Niigata University of Health and Welfare, Niigata 950-3198, Japan
p. School of Archaeology and Anthropology, College of Arts and Social Sciences, Australian National University, Canberra, ACT 2601, Australia
q. Department of Anatomy, Sapporo Medical University, Sapporo, Hokkaido 060-8556, Japan

1. H.-c.H., Z.D., and H.M. contributed equally to this work.
2. To whom correspondence may be addressed. Email:
   hsiao-chun.hung@anu.edu.au
   zhenhuadeng@pku.edu.cn
   hiromura@sapmed.ac.jp

This PDF file includes:

Supplementary Text
Figs. S1 to S11
Tables S1 to S5
References (1 to 97)

Supporting Information Text

Cut marks on bones and the question of whether "intentional dismemberment" occurred prior to burial. Some burials at Dingsishan had their skulls curiously inserted into their thoracic cavities, which had in turn been detached from the rest of the body. Another unusual burial practice involved placing the lower legs into forearm positions, connected at the elbow joints (Main text Fig. 10; *SI Appendix* Fig. S5). At Huiyaotian, burial M56 (Fig. 10: middle) initially appeared to be crouched and placed on its back, but closer examination revealed that the trunk, including the chest, neck, and skull, were positioned face down, whereas the sacrum and pelvis were oriented facing upwards. This anatomical arrangement suggests that the body was likely disarticulated and entirely twisted over at the location of the lumbar vertebrae.

Huiyaotian burial M29 had its knee joint, the distal end of the femur, and the proximal end of the tibia all positioned adjacent to the pelvis, these again being anatomically impossible orientations (Fig. 10: left). Even more mysteriously, Huiyaotian burial M15 (Fig. 10: right) was compacted into a block formation with its tightly packed ribs adjacent to the skull, although the lower limbs maintained some anatomical connections at the joints, and many finger and toe bones were also in their anatomical positions. There are further peculiarities with some of the squatting burials at Huiyaotian, in which bundles of long bones were placed upright in the burial pits, resembling tightly bundled firewood. In a number of cases, curiously, such bundles lacked some ribs and/or vertebrae (1).

In most of these burials, the majority of the skeletal remains were articulated in their correct anatomical positions, suggesting that the anatomically misplaced bones were either repositioned (sometimes incorrectly) in attempts to recreate their original anatomical locations.

Among the human skeletons examined from Huiyaotian (67 males, 26 females, and 7 individuals of undetermined sex), cut marks were identified on the bones of 19 individuals (2). They were each recorded and grouped into 47 sets based on spatial proximity and alignment, with closely spaced marks likely produced in a single continuous motion considered as one set. Lengths, widths, and locations of each cut mark and each set are detailed in Table S5. In terms of location, cut marks were recorded on the humerus (42%), femur (27%), tibia (11%), ulna (11%), fibula (4%), and patella (4%). Most were made near the epiphysis (86%), with only a few on the diaphysis (14%). Among the epiphyseal cut marks, the elbow joint exhibited the highest frequency, followed by the knee, hip, and ankle joints (Figs. S6 and S7) (2).

In addition, among the 26 bones with cut marks observed at Huiyaotian, two femora and three tibia show evidence for defleshing (Figs. S6 and S8).

Supplementary Figures

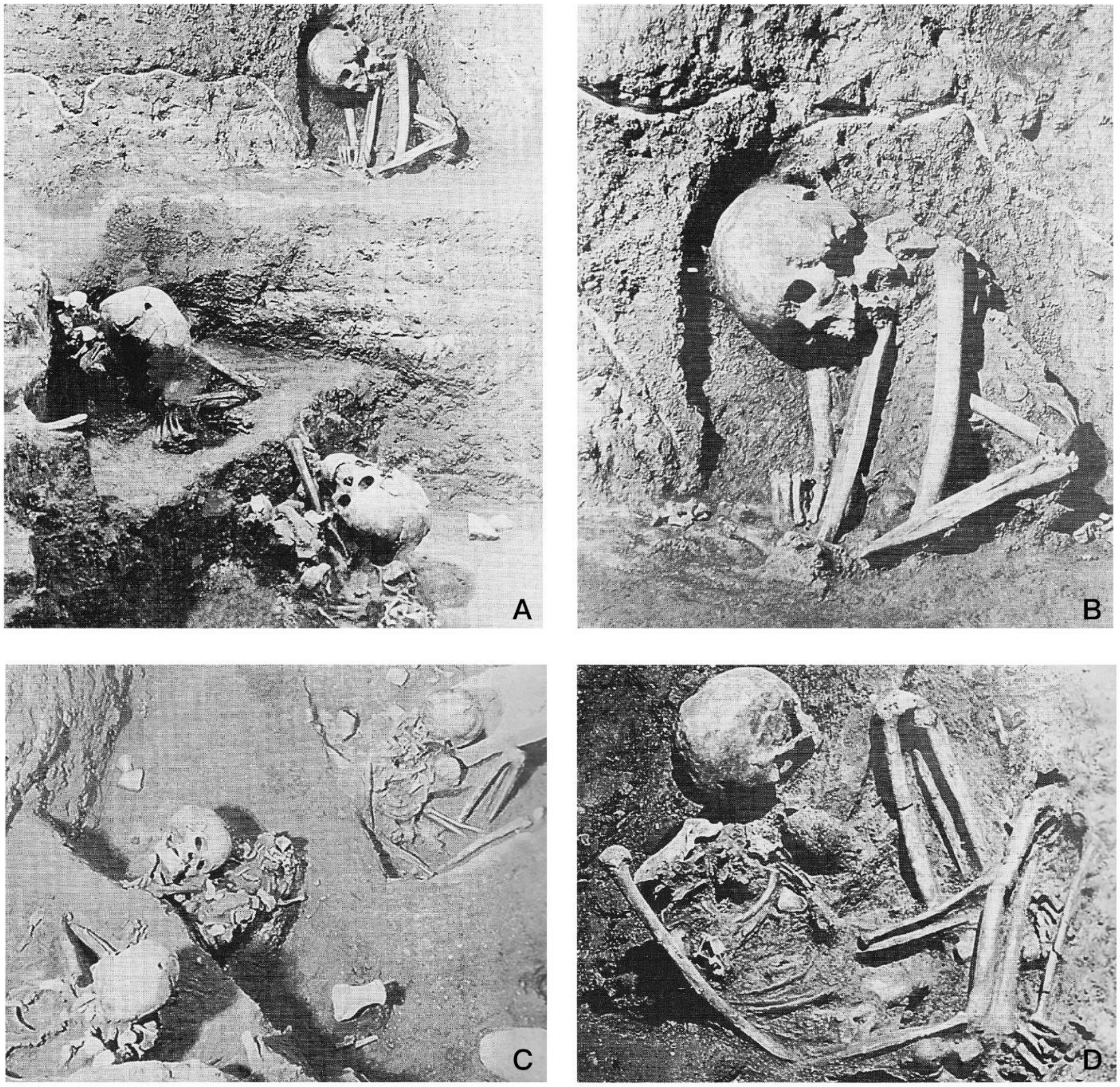

Fig. S1. Human burials exposed in Zengpiyan Cave (ca. 12,000-7,000 cal. BP), Guangxi, southern China, excavated in 1973.

(**A**) Three human burials exposed in excavation square BT2, viewed from the east; (**B**) Burial BT2M1 in a hyper-flexed position; (**C**) The three burials viewed from the south; (**D**) Burial BT2M5 in a flexed position, though not as tightly flexed as BT2M1 (3).

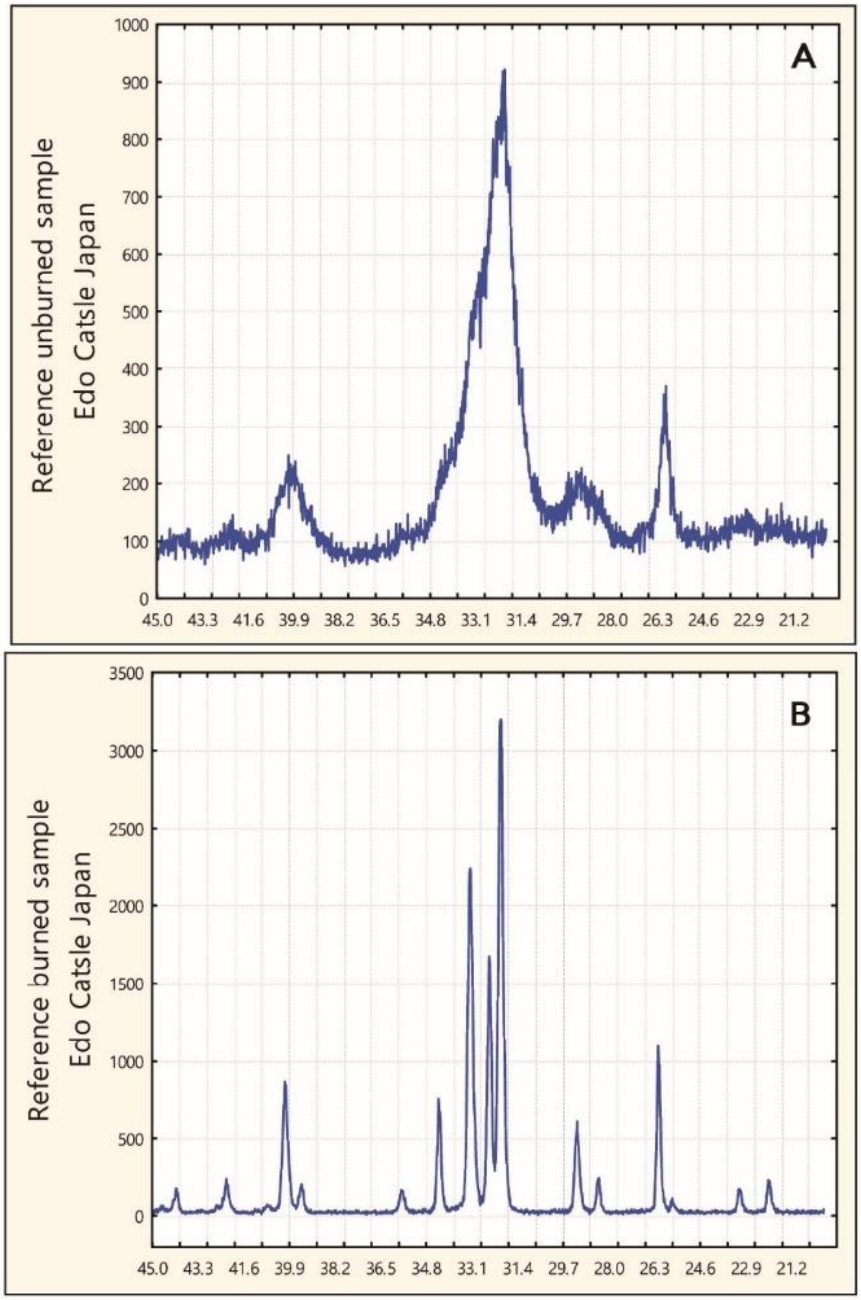

Fig. S2. X-ray diffraction patterns of the two control samples from the Early Modern Era, Edo Castle, Tokyo.

(**A**) The result for the unburned sample; (**B**) The result for the intentionally burned sample. Both results align with expectations, based on a previous study (4).

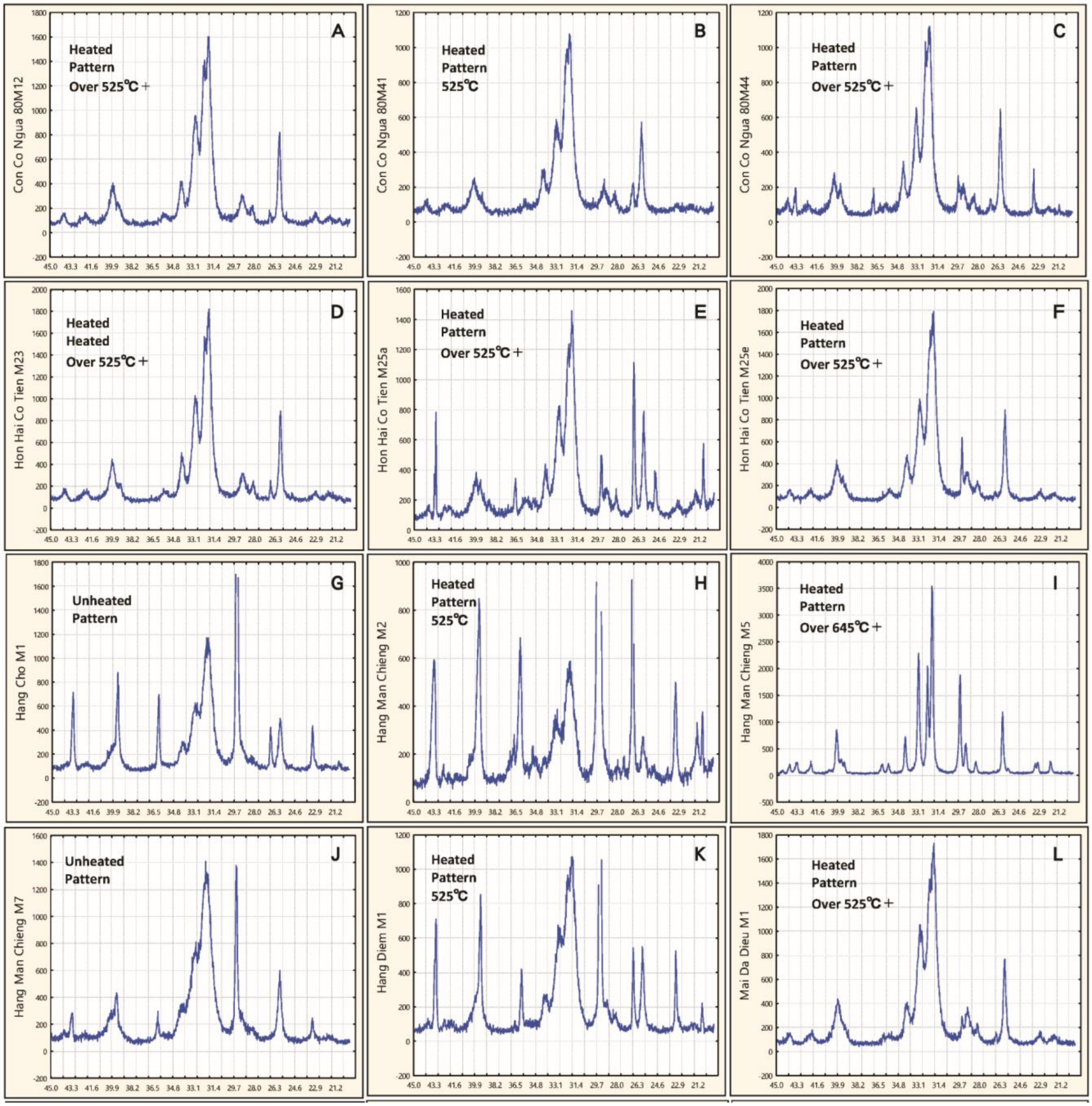

Fig. S3-1. X-ray diffraction patterns for the 20 studied samples.

(**A**) Con Co Ngua: 80M12; (**B**) Con Co Ngua: 80M41; (**C**) Con Co Ngua: 80M44; (**D**) Hon Hai Co Tien: M23; (**E**) Hon Hai Co Tien: M25a; (**F**) Hon Hai Co Tien: M25e; (**G**) Hang Cho: M1; (**H**) Hang Mang Chieng: M2; (**I**) Hang Mang Chieng: M5; (**J**) Hang Mang Chieng: M7; (**K**) Hang Diem: M1; (**L**) Mai Da Dieu: M1.

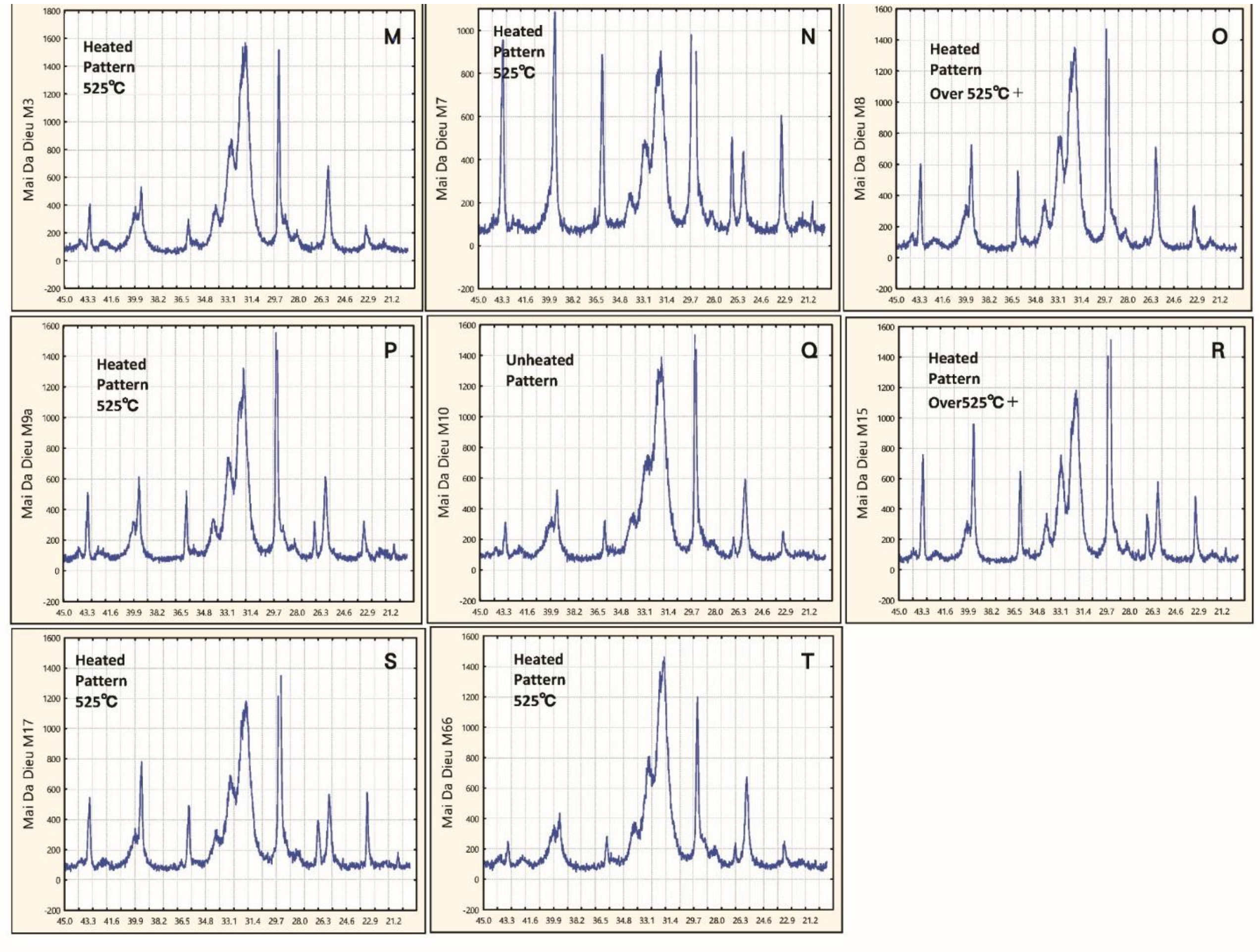

Fig. S3-2. X-ray diffraction patterns for the 20 studied samples.

(**M**) Mai Da Dieu: M3; (**N**) Mai Da Dieu: M7; (**O**) Mai Da Dieu: M8; (**P**) Mai Da Dieu: M9a; (**Q**) Mai Da Dieu: M10; (**R**) Mai Da Dieu: M15; (**S**) Mai Da Dieu: M17; (**T**) Mai Da Dieu: M66.

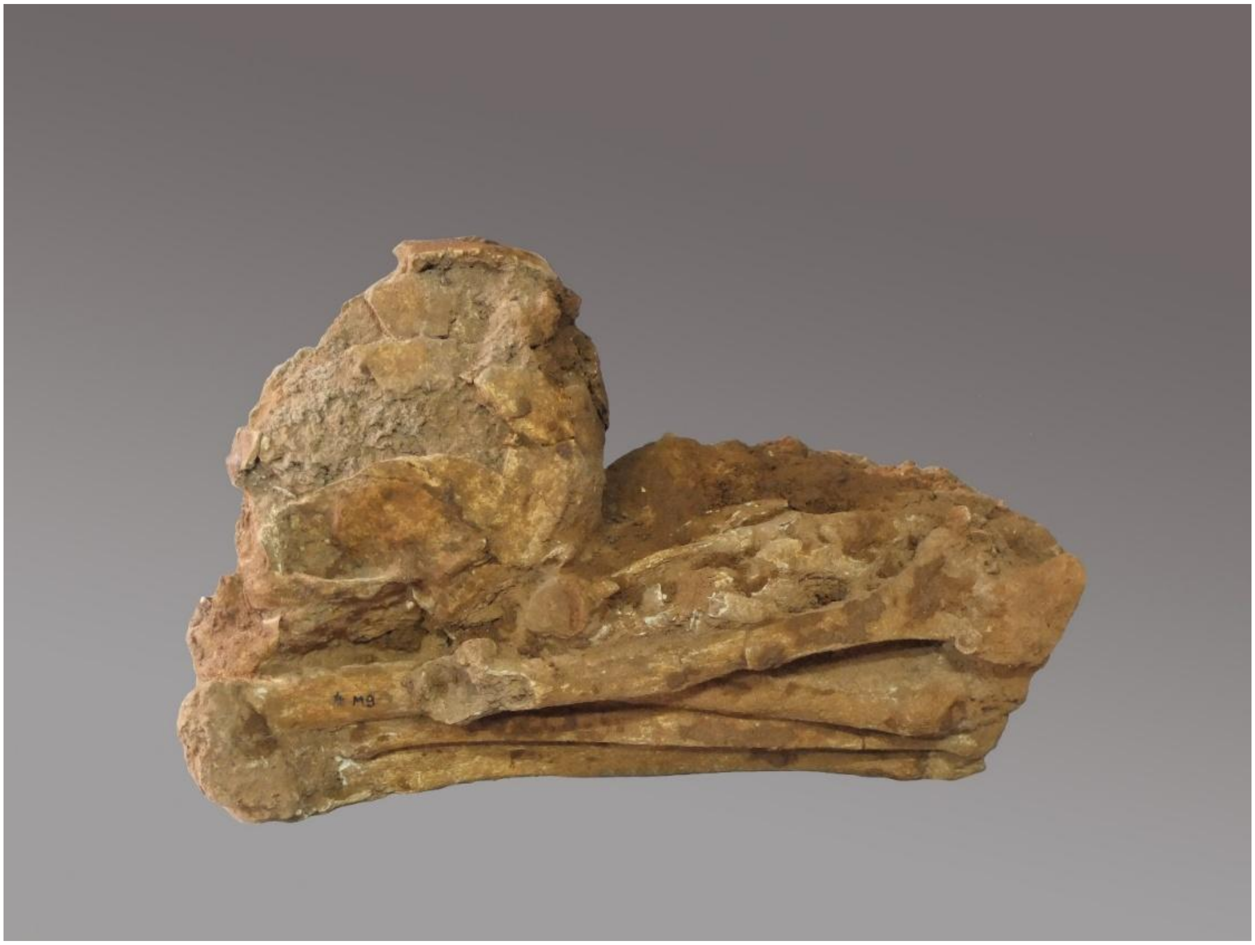

Fig. S4. Burial M9 from Huiyaotian, Guangxi, southern China.

At Huiyaotian and other sites such as Con Co Ngua in northern Vietnam, we observed that several cadavers were tightly flexed, likely bundled intentionally to achieve a compact form, and interred in a vertical posture. For instance, the burial position of Huiyaotian M9 resembles that of the hyper-flexed smoked mummy from Papua shown in Fig. 8 (A).

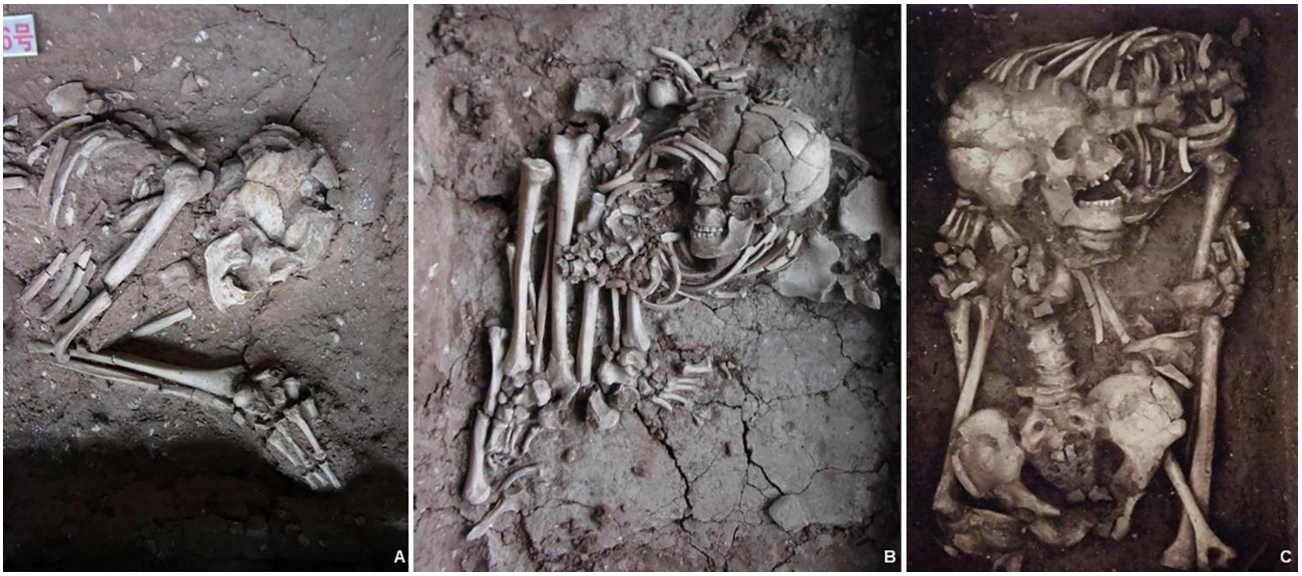

Fig. S5. Examples of burials from Dingsishan, Guangxi in southern China, previously suspected to be dismembered.

These three burials exhibit unusual anatomical positioning or the absence of certain bones: (**A**) foreleg and foot bones located in the forearm position; (**B**) and (**C**) heads positioned within the chest area (courtesy the Dingsishan Site Museum).

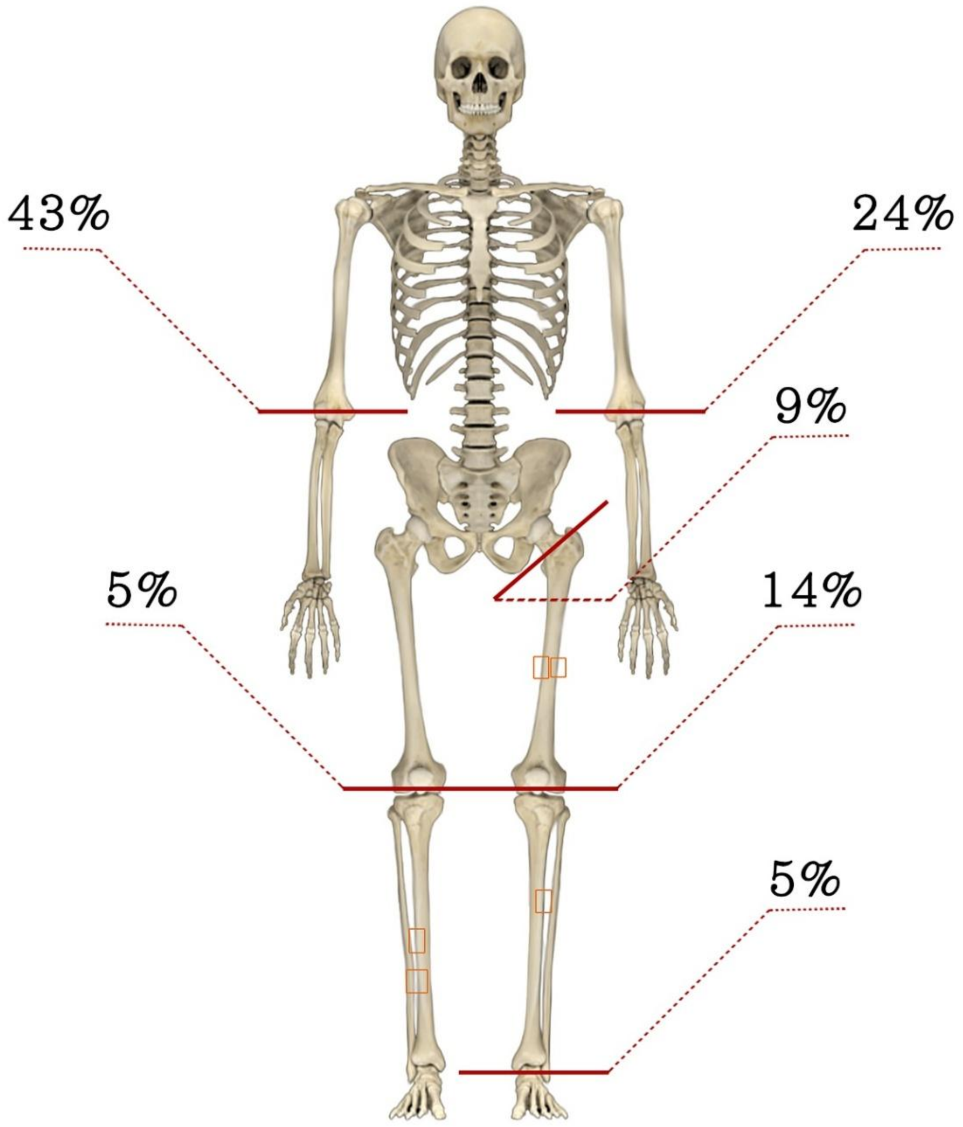

Fig. S6. Diagram showing the percentages of cut marks recorded different joints from the Huiyaotian sample of 19 individuals (excavated in 2016), Guangxi, southern China.

The small rectangles indicate additional cut marks on long bones that are considered to be from flesh removal, mostly from the femur and tibia (2).

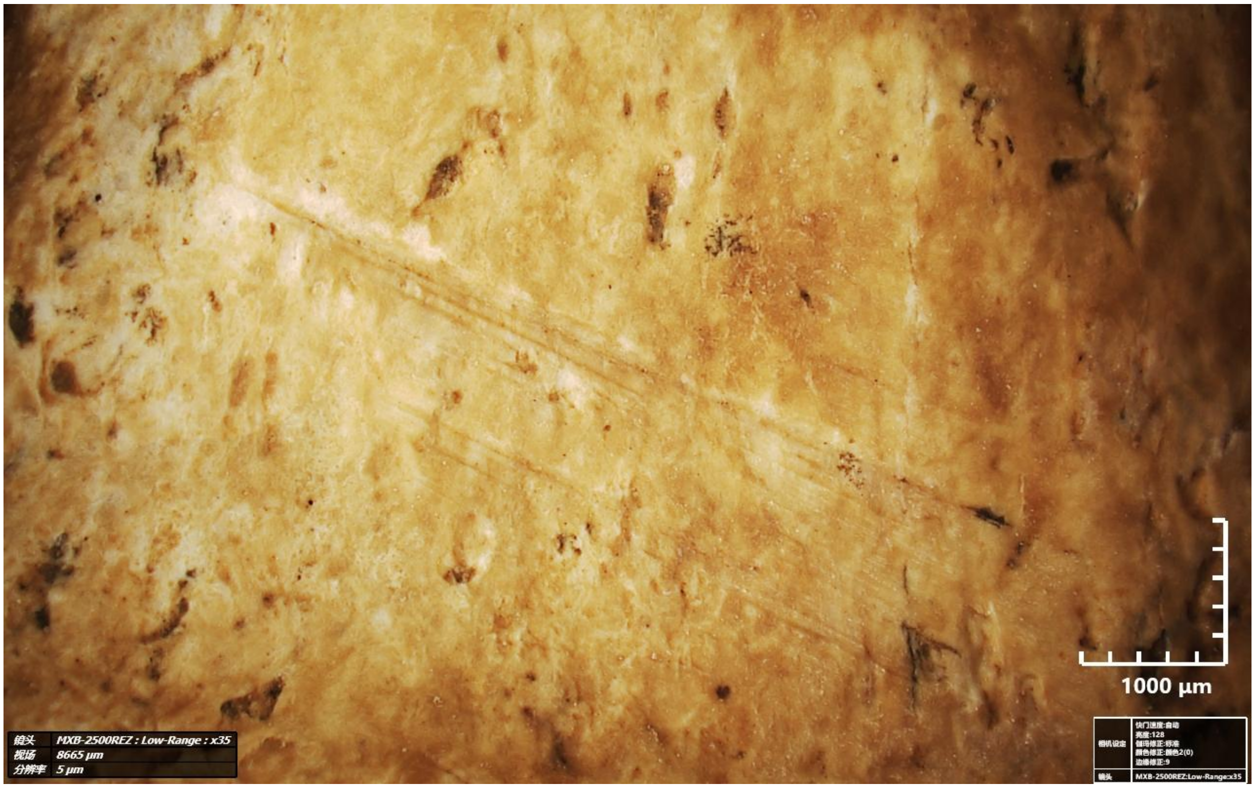

Fig. S7. Microscopic view of cut marks identified on a human bone from Huiyaotian, Guangxi, southern China.

This image shows the morphology of cut marks on specimen M53-H:1 (excavated in 2016). The marks are located near the epiphyseal region of the right humerus and are interpreted as evidence of disarticulation. A scale bar is included in the image (2).

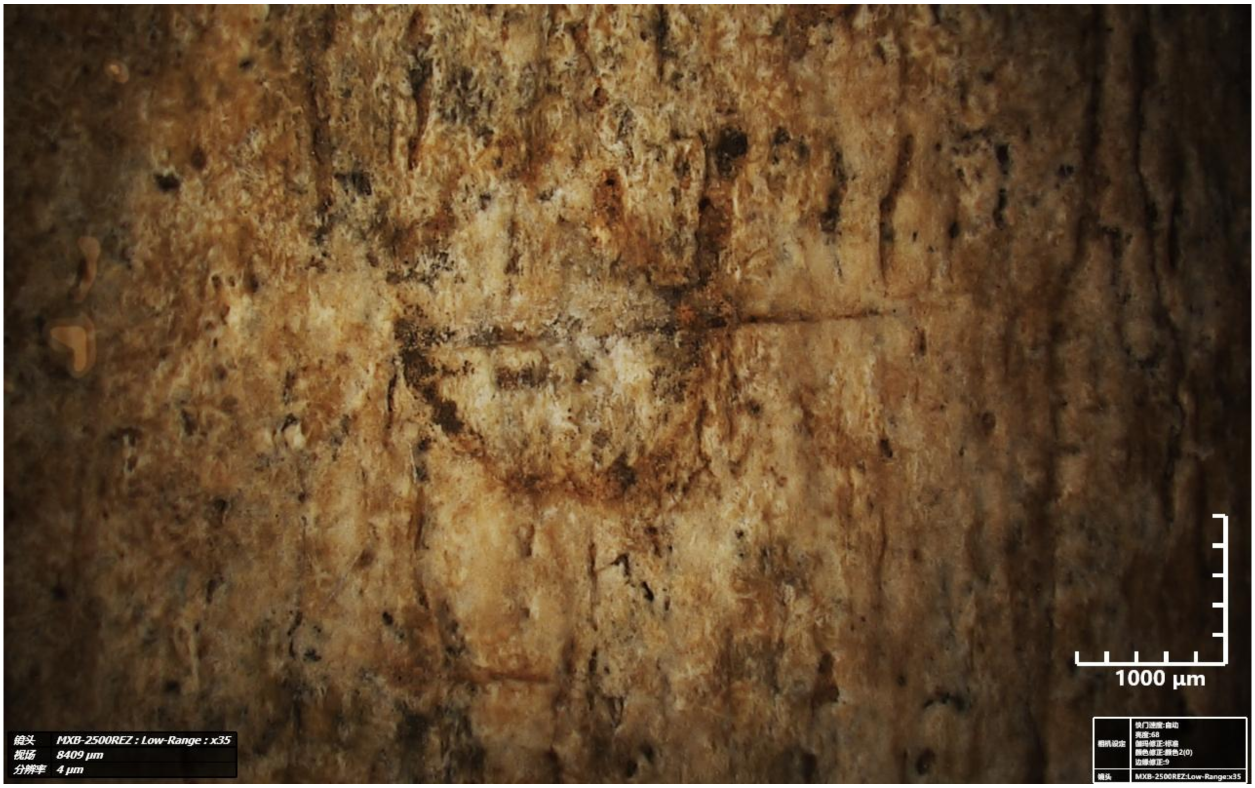

Fig. S8. Microscopic view of a cut mark identified on a human bone from Huiyaotian, Guangxi, southern China.

This image shows the morphology of the cut mark on specimen M16-F:2 (excavated in 2016). The mark is located near the diaphyseal region of the left femur and is interpreted as evidence of defleshing activity. A scale bar is included in the image (2).

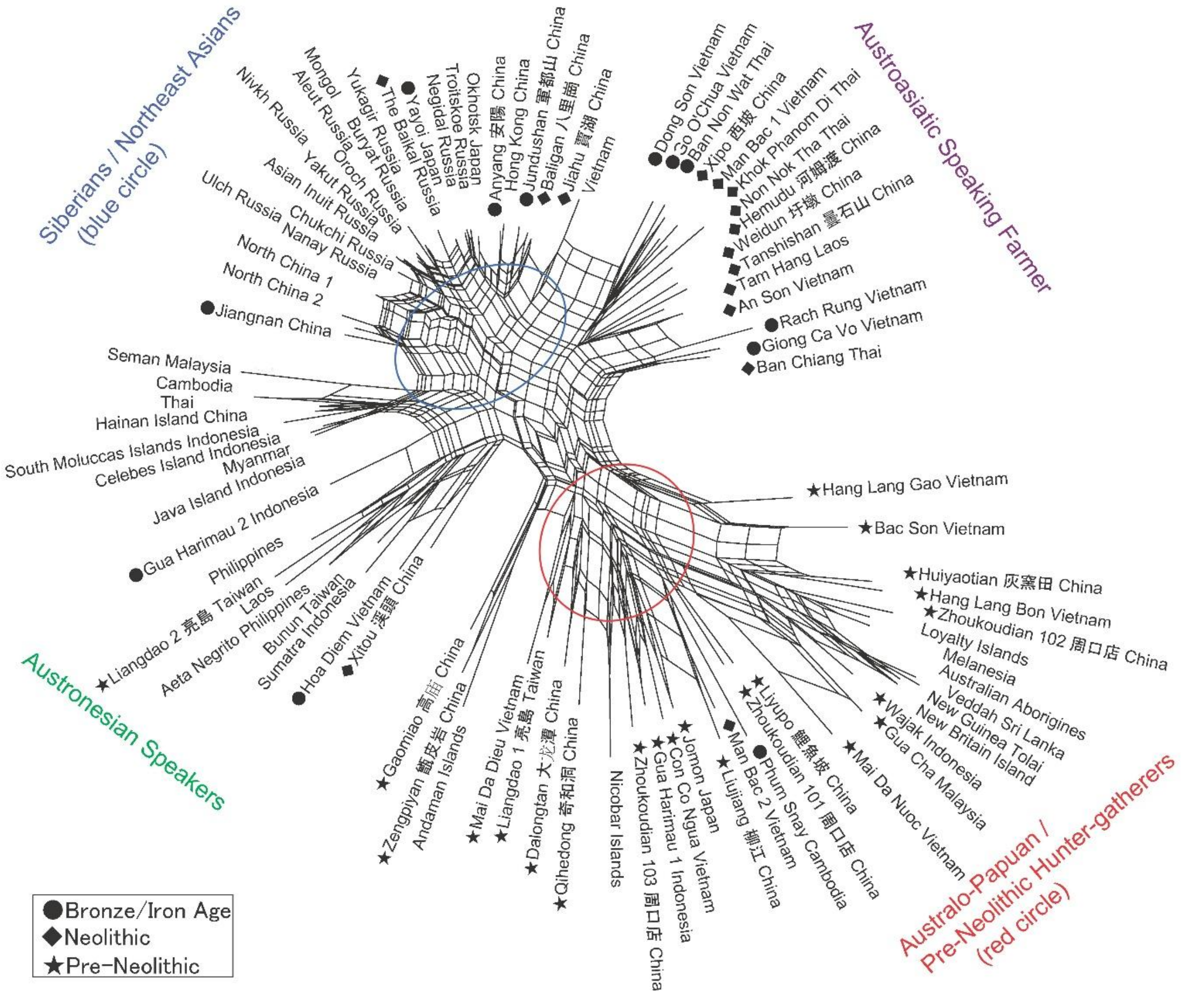

Fig. S9. Patterns in craniofacial morphology in a sample of ancient and modern crania in eastern Asia and western Oceania.

This figure comprises a Phoenix network map that was drawn using Neighbor Net Split analysis based on 16 morphometric measurements. Pre-Neolithic Southeast Asian hunter-gatherers are represented mostly by specimens from cave sites with Hoabinhian lithic associations. This Phoenix map reveals that most Pre-Neolithic individuals shared cranial characteristics with ancient and recent Australo-Papuans in Australia and New Guinea. These traits continued into later Dabutian hunter-gatherer contexts with pottery and ground stone axes, as at the shell midden sites of Con Co Ngua (Vietnam), Huiyaotian, and Liyupo (southern China). The pre-Neolithic hunter-gatherers buried in the cave of Gua Harimau (Sumatra, Indonesia) also share close affinities with ancient and recent Australo-Papuans (5).

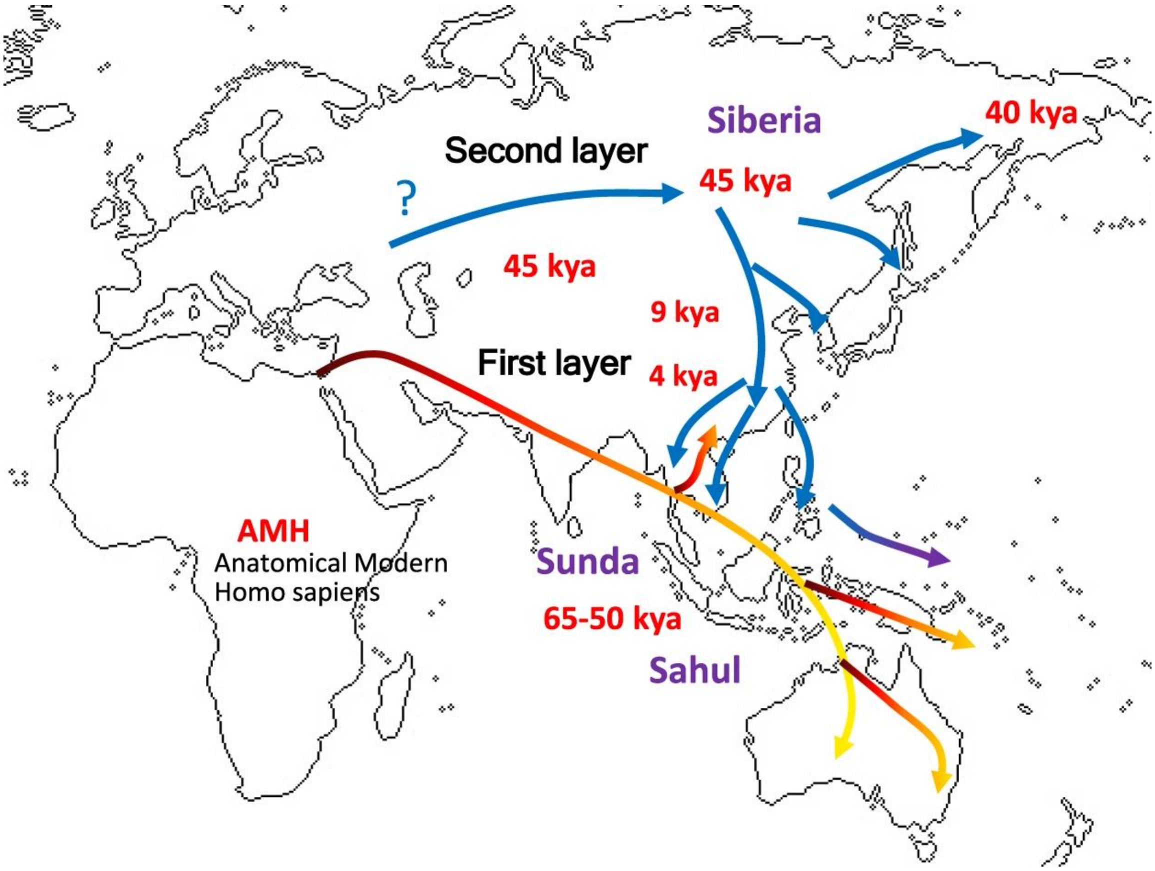

Fig. S10. The two-layer model for anatomically modern human (AMH) population movements across eastern Eurasia.

The southern and northern major migration contexts for early *Homo sapiens* dispersal towards eastern Asia may have been separated by mountain ranges and deserts in central Asia. The southern route followed the coastal rim of the Indian subcontinent and continued onwards into the ancient Pleistocene continent of Sahul by at least 50 kya. This southern migration was associated with a continuation of the Epipalaeolithic Hoabinhian pebble and flake tool complex, presumably a direct descendant of the Middle Palaeolithic industries introduced by the initial modern human inhabitants of this region. The northern route of early *Homo sapiens* dispersal is less clear, but in our scenario, it involved migration from western Eurasia across Siberia around 45 kya. ThePL descendants of this northern migration domesticated millet and rice after 9 kya in the Yellow and Yangtze valleys, and migrated south with their food producing economies to reach northern Mainland Southeast Asia by ~5 kya (5).

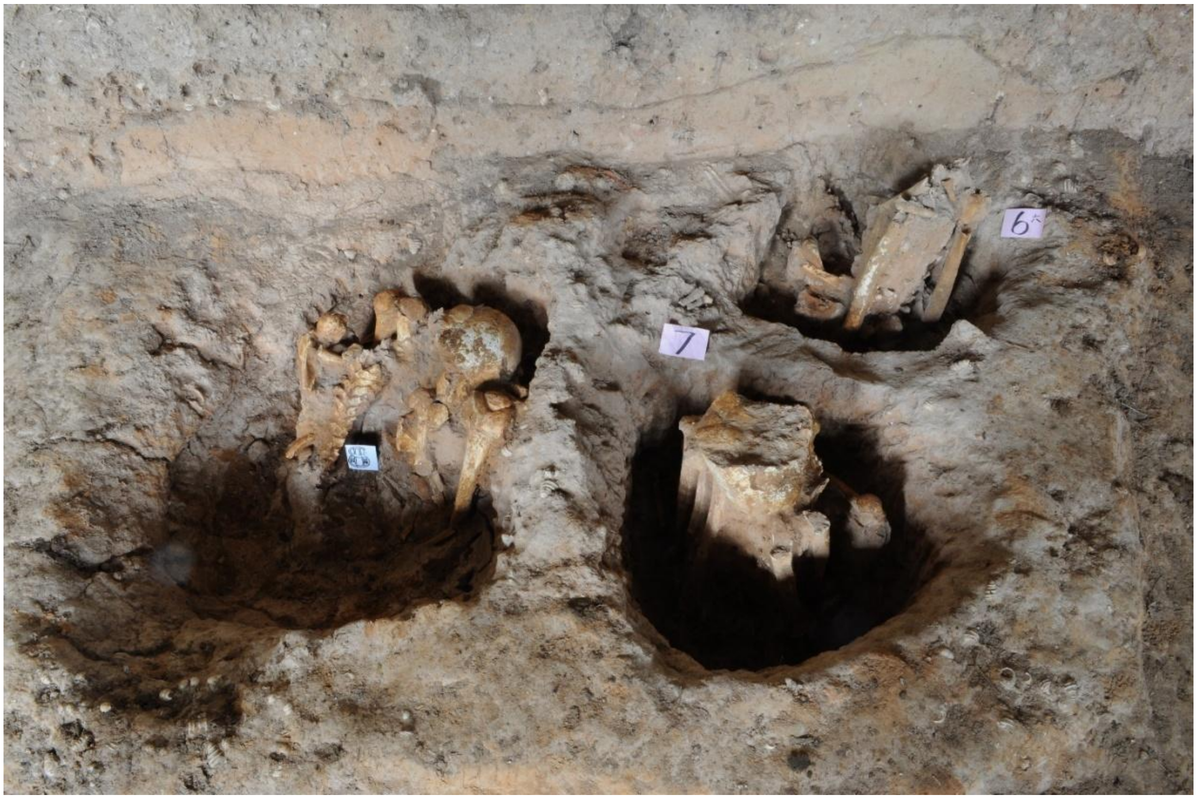

Fig. S11. Burials M6, M7, and M8 at the Huiyaotian site, Guangxi, southern China.

This image shows a corner of the excavation area at Huiyaotian, excavated in 2011. The three burials—M6 (female aged 40 to 60 y), M7 (a middle-aged adult female), and M8 (an adult male) — were all found in a squatting position.

Supplementary Tables

Table S1. Pre-Neolithic hunter-gatherer sites in Southeastern Asia exhibiting flexed, sitting, or squatting burial postures.

These 95 sites are located in southern China, Vietnam, Laos, the Philippines, Thailand, Malaysia, and Indonesia, and date to the terminal Pleistocene and the Early to Middle Holocene, within pre-agricultural contexts.

Site No. on Fig. 1	Site name	Longitude	Latitude	Reference
1	Gaomiao, Hunan	110° 4' 22.8″ E	27° 12' 46.8″ N	6
2	Niupodong, Guizhou	106° 27' 6.92″ E	26° 21' 57.04″ N	7
3	Zhaoguodong, Guizhou	106° 20' 48.62″ E	26° 22' 45.25″ N	8
4	Qihedong, Fujian	117° 23' 29.69″ E	25° 18' 0.97″ N	9
5	Daowei 1, Taiwan Strait	120° 13' 37.23″ E	26° 20' 42.82″ N	10
6	Xiaoma, Taiwan	121° 18' 25.23″ E	22° 58' 47.05″ N	11
7	Jiaoziyan, Guangxi	110° 14' 46.99″ E	25° 15' 37.1″ N	12
8	Zengpiyan, Guangxi	110° 16' 59.92″ E	25° 12' 47.94″ N	3
9	Miaoyan, Guangxi	110° 20' 37.44″ E	25° 5' 37.01″ N	13
10	Dayan, Guangxi	110° 12' 1.61″ E	25° 1' 12.53″ N	14
11	Xingyi, Yunnan	102° 49' 7.96″ E	24° 11' 0.43″ N	15
12	Baida, Guangxi	106° 14' 47.96″ E	23° 57' 40.03″ N	16
13	Gexinqiao, Guangxi	106° 32' 54.32″ E	23° 53' 15.98″ N	17
14	Beidaling, Guangxi	108° 24' 44.99″ E	24° 2' 22.09″ N	18
15	Liyuzui, Guangxi	109° 24' 48.49″ E	24° 16' 39.35″ N	19
16	Fengyan , Guangxi	109° 24' 59.89″ E	24° 14' 5.41″ N	20
17	Huangmenyan, Guangdong	113° 51' 10.69″ E	24° 11' 23.22″ N	8

Site No. on Fig. 1	Site name	Longitude	Latitude	Reference
18	Xiankezhou, Guangdong	112° 36' 24.43″ E	23° 10' 48.94″ N	21
19	Baxun, Guangxi	108° 3' 49.09″ E	23° 9' 45.93″ N	22
20	Liyupo, Guangxi	107° 57' 40.17″ E	23° 8' 48.93″ N	23, 24
21	Changtang, Guangxi	108° 39' 1.73″ E	22° 50' 8.19″ N	22
22	Lingwu, Guangxi	108° 31' 49.27″ E	22° 47' 23.01″ N	25
23	Huiyaotian, Guangxi	108° 25' 53.75″ E	22° 47' 15.68″ N	24, 26
24	Qingshan, Guangxi	108° 24' 4.42″ E	22° 46' 24.0″ N	22
25	Dingsishan, Guangxi	108° 29' 41.04″ E	22° 44' 55.43″ N	27
26	Qiujiang, Guangxi	108° 53' 11.71″ E	22° 43' 41.63″ N	28
27	Xijin, Guangxi	109° 14' 48.93″ E	22° 38' 49.72″ N	29
28	Ganzao, Guangxi	107° 54' 3.84″ E	22° 37' 38.72″ N	22
29	Hecun, Guangxi	107° 29' 5.28″ E	22° 29' 44.61″ N	30
30	Jiangbian, Guangxi	107° 29' 41.46″ E	22° 29' 10.12″ N	30
31	Chongtang, Guangxi	107° 21' 24.91″ E	22° 26' 53.06″ N	30
32	Lang Cuom, Lang Son	104° 56' 00″ E	22° 19' 00″ N	31
33	Mai Da Nguom, Thai Nguyen	105° 53' 9.19″ E	21° 47' 42.71″ N	32
34	Pho Binh Gia, Lang Son	106° 22' 00″ E	21° 57' 00″ N	31
35	Hang Doi, Lang Son	106°11' 3.57" E	21°47' 22.85" N	32
36	Dong Thuoc, Lang Son	106° 22' 59.88" E	21° 31' 59.88" N	31
37	Hang To 1, Son La	103° 39' 9.87" E	21° 38' 15.11" N	32
38	Hon Hai Co Tien, Quang Ninh	107° 5' 39.06″ E	20° 57' 4.76″ N	33
39	Cai Beo, Hai Phong	107° 3' 23.7″ E	20° 43' 40.37″ N	34

Site No. on Fig. 1	Site name	Longitude	Latitude	Reference
40	Liyudun, Guangdong	109° 43' 9.03″ E	21° 0' 46.53″ N	35
41	Dong Can, Hoa Binh	105° 23' 58.99″ E	20° 48'55.99″ N	32, 36
42	Du Sang , Hoa Binh	105° 26' 50.43″ E	20° 48' 25.17″ N	37
43	Lang Gao, Hoa Binh	106° 14' 00″ E	21° 38' 00″ N	38
44	Hang Cho, Hoa Binh	105° 39′ 08″ E	20° 50′ 07″ N	39
45	Hang Muoi, Hoa Binh	105° 18′ 00″ E	20° 36′ 00″ N	36, 40
46	Xom Trai, Hoa Binh	105° 24' 37.75″ E	20° 29' 43.24″ N	41
47	Hang Mang Chieng, Ninh Binh	105° 35' 34.79″ E	20° 19' 35.98″ N	42-44
48	Hang Con Moong, Thanh Hoa	105° 37' 25.29″ E	20° 15' 50.71″ N	45
49	Hang Diem, Ninh Binh	105° 55' 56.6″ E	20° 12' 43.8″ N	42, 46
50	Mai Da Dieu, Thanh Hoa	105° 9' 43.2″ E	20° 14' 34.8″ N	36, 47
51	Mai Da Nuoc, Thanh Hoa	105° 16' 20″ E	20° 24' 30″ N	48
52	Hang Lang Bon, Thanh Hoa	105° 28' 37.66" E	20° 11' 2.77" N	49,50
53	Da But, Thanh Hoa	105° 43' 0.31″ E	20° 1' 31.25″ N	51
54	Con Co Ngua, Thanh Hoa	105° 45' 21.55″ E	19° 59' 55.38″ N	52
55	Banyan Valley Cave, Mae Hong Son	98° 12' 0.0″ E	19° 38' 60.0″ N	53
56	Spirit Cave, Mae Hong Son	98° 5' 60.0″ E	19° 36' 0.0″ N	54
57	Tham Lod, Mae Hong Son	98° 17' 60.0″ E	19° 33' 18.0″ N	55
58	Ban Rai, Mae Hong Son	98° 12' 0.0″ E	19° 33' 0.0″ N	56
59	Doi Pha Kan, Lampang	99° 27' 58.32″ E	18° 16' 10.2″ N	57
60	Ban Tha Si, Lampang	99° 27' 35.28″ E	18° 15' 55.8″ N	58
61	Quynh Van, Nghe An	105° 39' 34.12″ E	19° 12' 4.63″ N	59

Site No. on Fig. 1	Site name	Longitude	Latitude	Reference
62	Pha Phen, Bolikhamsay	104° 33' 54.79″ E	18° 20' 55.95″ N	60
63	Yingdun, Hainan	109° 43' 19.07″ E	18° 18' 18.87″ N	61
64	Bau Du, Quang Nam	108° 30' 18.66″ E	15° 31' 3.45″ N	62-64
65	Krong No, Dak Nong	107° 48' 51.62″ E	12° 14' 52.8″ N	65
66	Bubog-1, Ilin Island	121° 4' 30.72″ E	12° 6' 5.76″ N	66
67	Ille Cave, Palawan	119° 30' 23.07″ E	11° 11' 48.9″ N	67
68	Duyong Cave, Palawan	117° 58' 53.31″ E	9° 16' 48.01″ N	68
69	Sa'gung, Palawan	118° 2' 2.43″ E	9° 13' 29.35″ N	69
70	Moh Khiew, Krabi	98° 33' 17.74″ E	8° 5' 37.08″ N	70
71	Gua Kerbau, Malay Peninsular	100° 18' 55.1″ E	6° 23' 22.37″ N	71
72	Gua Kepah, Malay Peninsular	100° 25' 34.47″ E	5° 33' 34.17″ N	71-72
73	Gua Kajang, Malay Peninsula	100° 58' 52.55″ E	5° 7' 34.68″ N	73
74	Gua Teluk Kelawar, Malay Peninsular	100° 58' 34.85″ E	5° 7' 7.73″ N	72
75	Gua Gunung Runtuh, Malay Peninsular	100° 58' 40.44″ E	5° 7' 24.51″ N	72
76	Gua Peraling, Malay Peninsula	101° 44' 59.63″ E	5° 2' 5.12″ N	72
77	Gua Cha, Malay Peninsula	101° 45' 43.32″ E	5° 1' 35.13″ N	72
78	Niah Cave, Borneo	113° 47' 7.58″ E	3° 48' 2.22″ N	74-77
79	Kimanis, Borneo	117° 14' 37.68″ E	2° 16' 13.44″ N	78
80	Liang Tebo, Borneo	117° 19' 13.72″ E	1° 7' 22.37″ N	79
81	Keboboh Cave, Borneo	117° 16' 46.07″ E	1° 4' 18.83″ N	80
82	Gua Tengkorak, Borneo	115° 54' 46.68″ E	1° 48' 15.73″ S	81
83	Leang Panninge, Sulawesi	119° 56' 25.21″ E	4° 47' 6.55″ S	82

Site No. on Fig. 1	Site name	Longitude	Latitude	Reference
84	Cappalombo 1, Sulawesi	119° 34' 28.58″ E	5° 2' 34.53″ S	83
85	Gua Harimau, Sumatra	103° 33' 18.72″ E	4° 2' 33.54″ S	84-87
86	Gua Pawon, Java	107° 26' 10.32″ E	6° 49' 26.96″ S	88
87	Gua Braholo, Java	110° 45' 7.77″ E	7° 57' 9.68″ S	89-91
88	Song Tritis, Java	110° 45' 7.77″ E	7° 57' 9.68″ S	90
89	Song Terus, Java	110° 59' 4.19″ E	8° 7' 51.38″ S	89, 92
90	Song Keplek, Java	111° 0' 8.85″ E	8° 7' 58.24″ S	92
91	Gua Lawa, Java	111° 21' 38.38″ E	7° 48' 34.62″ S	92-93
92	Song Gentong, Java	111° 45' 28.56″ E	8°7'17.23″ S	92
93	Tron Bon Lei, Alor	124° 34' 0.62″ E	8° 25' 41.48″ S	94
94	Ratu Mali 2, Kisar	127° 12' 24.2″ E	8° 3' 58.89″ S	95
95	Liang Lembudu, Aru	134° 32' 27.93″ E	6° 7' 27.43″ S	96

Table S2. Samples subjected to X-ray diffraction (XRD) and Fourier-transform infrared spectroscopy (FTIR) analyses.

A total of 71 samples were analyzed using XRD and FTIR, including 69 from pre-Neolithic contexts and 2 from a pre-modern Japanese site (Edo Castle in Tokyo), which served as control samples. The locations and ages of these sites are listed below, with many featuring direct radiocarbon (C14) dating on human bones. (Notes: 1. The letter "M" indicates a burial, in both Chinese and Vietnamese sites. 2. An asterisk (*) indicates samples with direct radiocarbon (C14) dates.

Sample Number	Site Name	Site Location	Site Form	Data Storage	Context	Date
Major study samples (N=69)						
1	Gaomiao	Hongjiang City, Hunan Province, China	Open site, shell midden	Hongjiang City Museum, Hunan, China	M1	*6,659-6,464 cal. BP (Beta-328354)
2	Gaomiao	Hongjiang City, Hunan Province, China	Open site, shell midden	Hongjiang City Museum, Hunan, China	M2	*6,452-6,300 cal. BP (Beta-328353)
3	Gaomiao	Hongjiang City, Hunan Province, China	Open site, shell midden	Hongjiang City Museum, Hunan, China	M20	*6,752-6,640 cal. BP (Beta-333225)
4	Huiyaotian	Nanning City, Guangxi Zhuang Autonomous Region, China	Open site, shell midden	Nanning City Museum, Guangxi, China	M7	9,000-8,300 cal. BP<br><br>*Human tooth from Layer 4 of T1003: 9,030-8,975 cal. BP (Beta-429237)
5	Huiyaotian	Nanning City, Guangxi Zhuang Autonomous Region, China	Open site, shell midden	Nanning City Museum, Guangxi, China	M14	9,000-8,300 cal. BP
6	Huiyaotian	Nanning City, Guangxi Zhuang Autonomous Region, China	Open site, shell midden	Nanning City Museum, Guangxi, China	M18	9,000-8,300 cal. BP
7	Huiyaotian	Nanning City, Guangxi Zhuang Autonomous Region, China	Open site, shell midden	Nanning City Museum, Guangxi, China	M18	9,000-8,300 cal. BP

Sample Number	Site Name	Site Location	Site Form	Data Storage	Context	Date
8	Huiyaotian	Nanning City, Guangxi Zhuang Autonomous Region, China	Open site, shell midden	Nanning City Museum, Guangxi, China	M19	9,000-8,300 cal. BP
9	Huiyaotian	Nanning City, Guangxi Zhuang Autonomous Region, China	Open site, shell midden	Nanning City Museum, Guangxi, China	M19	9,000-8,300 cal. BP
10	Huiyaotian	Nanning City, Guangxi Zhuang Autonomous Region, China	Open site, shell midden	Nanning City Museum, Guangxi, China	M23	9,000-8,300 cal. BP
11	Huiyaotian	Nanning City, Guangxi Zhuang Autonomous Region, China	Open site, shell midden	Nanning City Museum, Guangxi, China	M27	9,000-8,300 cal. BP
12	Huiyaotian	Nanning City, Guangxi Zhuang Autonomous Region, China	Open site, shell midden	Nanning City Museum, Guangxi, China	M35	9,000-8,300 cal. BP
13	Huiyaotian	Nanning City, Guangxi Zhuang Autonomous Region, China	Open site, shell midden	Nanning City Museum, Guangxi, China	M36	9,000-8,300 cal. BP
14	Huiyaotian	Nanning City, Guangxi Zhuang Autonomous Region, China	Open site, shell midden	Nanning City Museum, Guangxi, China	M39	9,000-8,300 cal. BP
15	Huiyaotian	Nanning City, Guangxi Zhuang Autonomous Region, China	Open site, shell midden	Nanning City Museum, Guangxi, China	M55	9,000-8,300 cal. BP
16	Liyupo	Long'an County, Guangxi Zhuang Autonomous Region, China	Open site, shell midden	Guangxi Institute of Cultural Relic Protection and Archaeology, Nanning, China	M1	8,000-6,700 cal. BP
17	Liyupo	Long'an County, Guangxi Zhuang Autonomous Region, China	Open site, shell midden	Guangxi Institute of Cultural Relic Protection and Archaeology, Nanning, China	M13	8,000-6,700 cal. BP<br><br>*Nearby M12: 7,667-7,580 cal. BP

Sample Number	Site Name	Site Location	Site Form	Data Storage	Context	Date
						(IAAA-143260)
18	Liyupo	Long'an County, Guangxi Zhuang Autonomous Region, China	Open site, shell midden	Guangxi Institute of Cultural Relic Protection and Archaeology, Nanning, China	M16	8,000-6,700 cal. BP
19	Liyupo	Long'an County, Guangxi Zhuang Autonomous Region, China	Open site, shell midden	Guangxi Institute of Cultural Relic Protection and Archaeology, Nanning, China	M19	8,000-6,700 cal. BP
20	Liyupo	Long'an County, Guangxi Zhuang Autonomous Region, China	Open site, shell midden	Guangxi Institute of Cultural Relic Protection and Archaeology, Nanning, China	M23	8,000-6,700 cal. BP
21	Liyupo	Long'an County, Guangxi Zhuang Autonomous Region, China	Open site, shell midden	Guangxi Institute of Cultural Relic Protection and Archaeology, Nanning, China	M28	8,000-6,700 cal. BP
22	Liyupo	Long'an County, Guangxi Zhuang Autonomous Region, China	Open site, shell midden	Guangxi Institute of Cultural Relic Protection and Archaeology, Nanning, China	M28	8,000-6,700 cal. BP
23	Liyupo	Long'an County, Guangxi Zhuang Autonomous Region, China	Open site, shell midden	Guangxi Institute of Cultural Relic Protection and Archaeology, Nanning, China	M29	8,000-6,700 cal. BP
24	Liyupo	Long'an County, Guangxi Zhuang Autonomous Region, China	Open site, shell midden	Guangxi Institute of Cultural Relic Protection and Archaeology, Nanning, China	M29	8,000-6,700 cal. BP
25	Liyupo	Long'an County, Guangxi Zhuang Autonomous Region, China	Open site, shell midden	Guangxi Institute of Cultural Relic Protection and Archaeology, Nanning, China	M30	8,000-6,700 cal. BP
26	Liyupo	Long'an County, Guangxi	Open site, shell midden	Guangxi Institute of Cultural Relic Protection and	M30	8,000-6,700 cal. BP

Sample Number	Site Name	Site Location	Site Form	Data Storage	Context	Date
		Zhuang Autonomous Region, China		Archaeology, Nanning, China		
27	Liyupo	Long'an County, Guangxi Zhuang Autonomous Region, China	Open site, shell midden	Guangxi Institute of Cultural Relic Protection and Archaeology, Nanning, China	M35	*6,944-6,741 cal. BP (BA-120617)
28	Liyupo	Long'an County, Guangxi Zhuang Autonomous Region, China	Open site, shell midden	Guangxi Institute of Cultural Relic Protection and Archaeology, Nanning, China	M38	8,000-6,700 cal. BP
29	Cai Beo	Cat Ba Island, Hai Phong Province, Vietnam	Open site	Vietnam Institute of Archaeology (Vien Khao Co Hoc), Hanoi, Vietnam	81 CB	ca. 7,000-5,000 cal. BP
30	Hon Hai Co Tien	Ha Long City, Quang Ninh Province, Vietnam	Rock Shelter and open site	Vietnam Institute of Archaeology (Vien Khao Co Hoc), Hanoi, Vietnam	04M10	ca. 5,000-4,000 cal. BP
31	Hon Hai Co Tien	Ha Long City, Quang Ninh Province, Vietnam	Rock Shelter and open site	Vietnam Institute of Archaeology (Vien Khao Co Hoc), Hanoi, Vietnam	04M11b	ca. 5,000-4,000 cal. BP
32	Hon Hai Co Tien	Ha Long City, Quang Ninh Province, Vietnam	Rock Shelter and open site	Vietnam Institute of Archaeology (Vien Khao Co Hoc), Hanoi, Vietnam	21M(A)H1L5 M6	*4,846-4,624 cal. BP (SANU-72807)
33	Hon Hai Co Tien	Ha Long City, Quang Ninh Province, Vietnam	Rock Shelter and open site	Vietnam Institute of Archaeology (Vien Khao Co Hoc), Hanoi, Vietnam	21M(A)H1L6 M7b	ca. 5,000-4,000 cal. BP
34	Hon Hai Co Tien	Ha Long City, Quang Ninh Province, Vietnam	Rock Shelter and open site	Vietnam Institute of Archaeology (Vien Khao Co Hoc), Hanoi, Vietnam	21M(A)H1 M4b	ca. 5,000-4,000 cal. BP
35	Hon Hai Co Tien	Ha Long City, Quang Ninh Province, Vietnam	Rock Shelter and open site	Vietnam Institute of Archaeology (Vien Khao Co Hoc), Hanoi, Vietnam	21M(A)H1 M4d	ca. 5,000-4,000 BP
36	Hon Hai Co Tien	Ha Long City, Quang Ninh Province, Vietnam	Rock Shelter and open site	Vietnam Institute of Archaeology (Vien Khao Co Hoc), Hanoi, Vietnam	21M(A)H1 M4g	ca. 5,000-4,000 BP
37	Hon Hai Co Tien	Ha Long City, Quang Ninh Province, Vietnam	Rock Shelter and open site	Vietnam Institute of Archaeology (Vien Khao Co Hoc), Hanoi, Vietnam	21M(A)H1 M7c	*4,286-4,005 cal. BP (SANU-72806)
38	Hon Hai Co Tien	Ha Long City, Quang Ninh	Rock Shelter and open site	Vietnam Institute of Archaeology (Vien	M23	ca. 5,000-4,000

Sample Number	Site Name	Site Location	Site Form	Data Storage	Context	Date
		Province, Vietnam		Khao Co Hoc), Hanoi, Vietnam		cal. BP
39	Hon Hai Co Tien	Ha Long City, Quang Ninh Province, Vietnam	Rock Shelter and open site	Vietnam Institute of Archaeology (Vien Khao Co Hoc), Hanoi, Vietnam	M25a	ca. 5,000-4,000 cal. BP
40	Hon Hai Co Tien	Ha Long City, Quang Ninh Province, Vietnam	Rock Shelter and open site	Vietnam Institute of Archaeology (Vien Khao Co Hoc), Hanoi, Vietnam	M25e	ca. 5,000-4,000 cal. BP
41	Mai Da Dieu	Thanh Hoa Province, Vietnam	Cave site/ Epi-Hoabinhian	Vietnam Institute of Archaeology (Vien Khao Co Hoc), Hanoi, Vietnam	M1	ca. 8,000-4,000 cal. BP
42	Mai Da Dieu	Thanh Hoa Province, Vietnam	Cave site/ Epi-Hoabinhian	Vietnam Institute of Archaeology (Vien Khao Co Hoc), Hanoi, Vietnam	M1-bone	ca. 8,000-4,000 cal. BP
43	Mai Da Dieu	Thanh Hoa Province, Vietnam	Cave site/ Epi-Hoabinhian	Vietnam Institute of Archaeology (Vien Khao Co Hoc), Hanoi, Vietnam	M1-tooth	ca. 8,000-4,000 cal. BP
44	Mai Da Dieu	Thanh Hoa Province, Vietnam	Cave site/ Epi-Hoabinhian	Vietnam Institute of Archaeology (Vien Khao Co Hoc), Hanoi, Vietnam	M3	ca. 8,000-4,000 cal. BP
45	Mai Da Dieu	Thanh Hoa Province, Vietnam	Cave site/ Epi-Hoabinhian	Vietnam Institute of Archaeology (Vien Khao Co Hoc), Hanoi, Vietnam	M7	*4,291-4,006 cal. BP (OxA-35860) (97)
46	Mai Da Dieu	Thanh Hoa Province, Vietnam	Cave site/ Epi-Hoabinhian	Vietnam Institute of Archaeology (Vien Khao Co Hoc), Hanoi, Vietnam	M7	*4,291-4,006 cal. BP (OxA-35860) (97)
47	Mai Da Dieu	Thanh Hoa Province, Vietnam	Cave site/ Epi-Hoabinhian	Vietnam Institute of Archaeology (Vien Khao Co Hoc), Hanoi, Vietnam	M8	ca. 8,000-4,000 cal. BP
48	Mai Da Dieu	Thanh Hoa Province, Vietnam	Cave site/ Epi-Hoabinhian	Vietnam Institute of Archaeology (Vien Khao Co Hoc), Hanoi, Vietnam	M9a	ca. 8,000-4,000 cal. BP
49	Mai Da Dieu	Thanh Hoa Province, Vietnam	Cave site/ Epi-Hoabinhian	Vietnam Institute of Archaeology (Vien Khao Co Hoc), Hanoi, Vietnam	M10	ca. 8,000-4,000 cal. BP
50	Mai Da Dieu	Thanh Hoa Province, Vietnam	Cave site/ Epi-Hoabinhian	Vietnam Institute of Archaeology (Vien Khao Co Hoc), Hanoi, Vietnam	M15	ca. 8,000-4,000 cal. BP

Sample Number	Site Name	Site Location	Site Form	Data Storage	Context	Date
51	Mai Da Dieu	Thanh Hoa Province, Vietnam	Cave site/ Epi-Hoabinhian	Vietnam Institute of Archaeology (Vien Khao Co Hoc), Hanoi, Vietnam	M17	ca. 8,000-4,000 cal. BP
52	Mai Da Dieu	Thanh Hoa Province, Vietnam	Cave site/ Epi-Hoabinhian	Vietnam Institute of Archaeology (Vien Khao Co Hoc), Hanoi, Vietnam	M17	ca. 8,000-4,000 cal. BP
53	Mai Da Dieu	Thanh Hoa Province, Vietnam	Cave site/ Epi-Hoabinhian	Vietnam Institute of Archaeology (Vien Khao Co Hoc), Hanoi, Vietnam	M66	ca. 8,000-4,000 cal. BP
54	Con Co Ngua	Thanh Hoa Province, Vietnam	Open site, shell midden	Vietnam Institute of Archaeology (Vien Khao Co Hoc), Hanoi, Vietnam	80CCN-M12E	6,700-6,200 cal. cal. BP
55	Con Co Ngua	Thanh Hoa Province, Vietnam	Open site, shell midden	Vietnam Institute of Archaeology (Vien Khao Co Hoc), Hanoi, Vietnam	80CCN-M41	6,700-6,200 cal. BP
56	Con Co Ngua	Thanh Hoa Province, Vietnam	Open site, shell midden	Vietnam Institute of Archaeology (Vien Khao Co Hoc), Hanoi, Vietnam	80CCN-M44	6,200-6,700 cal. BP
57	Con Co Ngua	Thanh Hoa Province, Vietnam	Open site, shell midden	Vietnam Institute of Archaeology (Vien Khao Co Hoc), Hanoi, Vietnam	80CCNM44-in_black	6,700-6,200 cal. BP
58	Con Co Ngua	Thanh Hoa Province, Vietnam	Open site, shell midden	Vietnam Institute of Archaeology (Vien Khao Co Hoc), Hanoi, Vietnam	80CCNM44-out_white	6,700-6,200 cal. BP
59	Hang Cho	Hoa Binh Province, Vietnam	Cave site	Vietnam Institute of Archaeology (Vien Khao Co Hoc), Hanoi, Vietnam	M1	*11,150-9,750 cal. BP (39)
60	Hang Diem	Hoa Binh Province, Vietnam	Cave site	Vietnam Institute of Archaeology (Vien Khao Co Hoc), Hanoi, Vietnam	M1	10,000-5,000 cal. BP
61	Hang Mang Chieng	Hoa Binh Province, Vietnam	Cave site	Vietnam Institute of Archaeology (Vien Khao Co Hoc), Hanoi, Vietnam	M5	18,000-5,000 cal. BP
62	Hang Mang Chieng	Hoa Binh Province, Vietnam	Cave site	Vietnam Institute of Archaeology (Vien Khao Co Hoc), Hanoi, Vietnam	M2-1	18,000-5,000 cal. BP
63	Hang Mang Chieng	Hoa Binh Province, Vietnam	Cave site	Vietnam Institute of Archaeology (Vien Khao Co Hoc), Hanoi, Vietnam	M2-2	18,000-5,000 cal. BP

Sample Number	Site Name	Site Location	Site Form	Data Storage	Context	Date
64	Hang Mang Chieng	Hoa Binh province, Vietnam	Cave site	Vietnam Institute of Archaeology (Vien Khao Co Hoc), Hanoi, Vietnam	M2-3	18,000-5,000 cal. BP
65	Hang Mang Chieng	Hoa Binh province, Vietnam	Cave site	Vietnam Institute of Archaeology (Vien Khao Co Hoc), Hanoi, Vietnam	M7-in_yellow	18,000-5,000 cal. BP
66	Hang Mang Chieng	Hoa Binh province, Vietnam	Cave site	Vietnam Institute of Archaeology (Vien Khao Co Hoc), Hanoi, Vietnam	M7-out_black	18,000-5,000 cal. BP
67	Hang Mang Chieng	Hoa Binh province, Vietnam	Cave site	Vietnam Institute of Archaeology (Vien Khao Co Hoc), Hanoi, Vietnam	M7-out_yellow	18,000-5,000 cal. BP
68	Gua Harimau	Ogan Komering Ulu (OKU) Regency, South Sumatra, Indonesia	Cave site	Si Pahit Lidah Museum, southern Sumatra, Indonesia	No. 57	3,689-3,482 cal. BP (BTN12019 Batan Lab Indonesia) (86)
69	Gua Harimau	Ogan Komering Ulu (OKU) Regency, South Sumatra, Indonesia	Cave site	Si Pahit Lidah Museum, southern Sumatra, Indonesia	No. 79	*3,819-3,568 cal. BP (Beta-452948)
Control samples (N=2)						
70	Edo Castle	Edo Castle, Tokyo, Japan	Open site	National Science Museum, Tokyo, Japan	No 1	ca. 300 cal. BP
71	Edo Castle	Edo Castle, Tokyo, Japan	Open site	National Science Museum, Tokyo, Japan	No 1	ca. 300 cal. BP

Table S3. Results of X-ray diffraction (XRD) analysis of 20 study samples and 2 control samples.

The 20 study samples exhibit varying degrees of exposure to heat, including temperatures exceeding 645°C, over 525°C, approximately 525°C, and three samples with uncertain results, likely due to temperatures being too low to be detected.

**Sample Number**	**Site Name**	**Context**	**Visible black-coloured burned bones of the skeleton**	**Examined bones**	**XRD Result**
Major study samples (N=20)					
38	Hon Hai Co Tien	M23	none	tibia	> 525°C
39	Hon Hai Co Tien	M25a	none	clavicle	> 525°C
40	Hon Hai Co Tien	M25e	none	unknown limb fragment	> 525°C
41	Mai Da Dieu	M1	none	talus	> 525°C
44	Mai Da Dieu	M3	pubis	pubis	~525°C
46	Mai Da Dieu	M7	ulna and radius	rib	~525°C
47	Mai Da Dieu	M8	none	upper limb fragment	> 525°C
48	Mai Da Dieu	M9a	none	rib	~525°C
49	Mai Da Dieu	M10	none	ulna	Unheated?
50	Mai Da Dieu	M15	none	skull	> 525°C
51	Mai Da Dieu	M17	right frontal bone, humerus, ulna, ribs, tibia, and metacarpal bones	metacarpal bone	~525°C
53	Mai Da Dieu	M66	none	unknown limb fragment	~525°C
54	Con Co Ngua	80CCN-M12E	none	skull	> 525°C
55	Con Co Ngua	80CCN-M41	distal end of left femur	left femur	~525°C
56	Con Co Ngua	80CCN-M44	none	unknown limb fragment	> 525°C
59	Hang Cho	M1	none	skull	Unheated?
60	Hang Diem	M1	none	rib	~525°C
61	Hang Mang Chieng	M5	foot bones	metatarsal bone	> 645°C
62	Hang Mang Chieng	M2-1	none	skull	~525°C
66	Hang Mang Chieng	M7-out_black	fibula (blackened due to heavy smoke? or sediment?)	fibula	Unheated?
Control samples (N=2)					
70	Edo Castle	No 1	unknown limbs	unknown limb fragments/ original unheated	Unheated

Sample Number	Site Name	Context	Visible black-coloured burned bones of the skeleton	Examined bones	XRD Result
71	Edo Castle	No 1	unknown limbs	unknown limb fragments/ intentional heated before XRD	> 645°C

Table S4. Results of FTIR analysis of the human bone sample.

Sixty-nine human bone samples from 11 pre-Neolithic sites were analyzed using Fourier-transform infrared spectroscopy (FTIR). The crystallinity index (CI) was calculated by summing the peak intensities at 603 cm^{-1} and 565 cm^{-1} and dividing the result by the intensity of the trough between them. Excluding the five samples with poor signal quality, the remaining 64 samples were classified into three groups based on their CI values: unheated (below 3.25), probably heated (3.25–3.8), and heated to temperatures exceeding 400°C (above 3.8). Additionally, an intentionally heated control sample from Edo Castle was analyzed and found to have been heated above 400°C.

**Sample Number**	**Site Name**	**Context**	**Visible black-coloured burned bones of the skeleton**	**Examined bones**	peak/trough intensity			CI	OH^- libration band at ca. 630 cm^{-1}	Group
					peak 603 cm^{-1}	peak 565 cm^{-1}	in-between trough			
Major study samples (n=69)										
1	Gaomiao	M1	none	rib	0.718	0.813	0.473	3.24	Null	Unheated
2	Gaomiao	M2	none	phalange	0.844	0.998	0.517	3.56	Null	Probably heated
3	Gaomiao	M20	none	unknown limb fragment	0.783	0.876	0.472	3.51	Null	Probably heated
4	Huiyaotian	M7	none	rib	0.349	0.41	0.203	3.74	Null	Probably heated
5	Huiyaotian	M14	none	right tibia	poor signal					
6	Huiyaotian	M18	right radius	left ulna	0.961	1.188	0.503	4.27	Weak	> 400°C
7	Huiyaotian	M18	right radius	left ulna	0.648	0.747	0.38	3.67	Null	Probably heated
8	Huiyaotian	M19	none	fibula	1.763	2.097	0.908	4.25	Obvious	> 400°C
9	Huiyaotian	M19	none	left mandible	poor signal					
10	Huiyaotian	M23	none	unknown limb fragment	0.746	0.81	0.446	3.49	Null	Probably heated
11	Huiyaotian	M27	distal end of humerus	right humerus	0.313	0.352	0.185	3.59	Null	Probably heated
12	Huiyaotian	M35	none	fibula	0.972	1.221	0.382	5.74	Null	> 400°C

Sample Number	Site Name	Context	Visible black-coloured burned bones of the skeleton	Examined bones	peak/trouph intensity			CI	OH^- libration band at ca. 630 cm^{-1}	Group
					peak 603 cm^{-1}	peak 565 cm^{-1}	in-between trouph			
13	Huiyaotian	M36	right ulna and radius, scapula, rib, distal end of the tibia, and distal end of the femur	left radius	0.945	1.027	0.6	3.29	Null	Probably heated
14	Huiyaotian	M39	none	fibula	0.888	1.066	0.437	4.47	Weak	> 400°C
15	Huiyaotian	M55	tibia and pelvis	right tibia	1.41	1.707	0.76	4.10	Null	> 400°C
16	Liyupo	M1	none	femur	0.904	1.005	0.527	3.62	Null	Probably heated
17	Liyupo	M13	none	rib	0.94	1.003	0.638	3.05	Null	Unheated
18	Liyupo	M16	none	rib	0.468	0.529	0.293	3.40	Null	Probably heated
19	Liyupo	M19	none	rib	0.79	0.94	0.454	3.81	Null	> 400°C
20	Liyupo	M23	none	fibula	1.309	1.418	0.859	3.17	Null	Unheated
21	Liyupo	M28	none	fibula	0.893	1.064	0.519	3.77	Null	Probably heated
22	Liyupo	M28	none	unknown limb fragment	1.324	1.341	0.945	2.82	Null	Unheated
23	Liyupo	M29	femur	rib	0.726	0.805	0.455	3.36	Null	Probably heated
24	Liyupo	M29	femur	left mandible	0.464	0.525	0.26	3.80	Null	> 400°C
25	Liyupo	M30	fibula	left fibula	0.443	0.499	0.252	3.74	Null	Probably heated
26	Liyupo	M30	fibula	left fibula	1.034	1.346	0.527	4.52	Obvious	> 400°C
27	Liyupo	M35	right frontal and zygomatic bones	rib	1.502	1.646	0.885	3.56	Null	Probably heated
28	Liyupo	M38	none	rib	0.698	0.763	0.44	3.32	Null	Probably heated
29	Cai Beo	81 CB	none	skull	0.461	0.496	0.206	4.65	Null	> 400°C

**Sample Number**	**Site Name**	**Context**	**Visible black-coloured burned bones of the skeleton**	**Examined bones**	peak/trouph intensity			CI	OH⁻ libration band at ca. 630 cm⁻¹	Group
					peak 603 cm^{-1}	peak 565 cm^{-1}	in-between trouph			
30	Hon Hai Co Tien	04 M10	none	unknown limb fragment	0.57	0.645	0.318	3.82	Null	> 400°C
31	Hon Hai Co Tien	04 M11b	none	unknown limb fragment	poor signal					
32	Hon Hai Co Tien	21 H1L5 M6	none	mandible	1.158	1.232	0.717	3.33	Null	Probably heated
33	Hon Hai Co Tien	21 M(A) H1L6M7b	none	unknown limb fragment	0.74	0.777	0.422	3.59	Null	Probably heated
34	Hon Hai Co Tien	21 M(A) H1M4b	none	tooth	0.922	0.912	0.659	2.78	Null	Unheated
35	Hon Hai Co Tien	21 M(A) H1M4d	none	unknown limb fragment	0.348	0.465	0.209	3.89	Null	> 400°C
36	Hon Hai Co Tien	21 M(A) H1M4g	none	unknown limb fragment	0.808	0.947	0.428	4.10	Null	> 400°C
37	Hon Hai Co Tien	21 M(A) H1M7c	none	skull	1.254	1.331	0.791	3.27	Null	Probably heated
38	Hon Hai Co Tien	M23	none	tibia	0.218	0.243	0.123	3.75	Null	Probably heated
39	Hon Hai Co Tien	M25a	none	clavicle	0.476	0.618	0.239	4.58	Weak	> 400°C
40	Hon Hai Co Tien	M25e	none	unknown limb fragment	0.0805	0.0905	0.0464	3.69	Weak	Probably heated
41	Mai Da Dieu	M1	none	talus	1.901	1.926	0.668	5.73	Obvious	> 400°C
42	Mai Da Dieu	M1-bone	in all limbs, but not in the vertebrae	skull	poor signal					
43	Mai Da Dieu	M1-tooth	in all limbs, but not in the vertebrae	tooth	0.0779	0.0825	0.0491	3.27	Null	Probably heated

Sample Number	Site Name	Context	Visible black-coloured burned bones of the skeleton	Examined bones	peak/trough intensity			CI	OH⁻ libration band at ca. 630 cm⁻¹	Group
					peak 603 cm^{-1}	peak 565 cm^{-1}	in-between trough			
44	Mai Da Dieu	M3	pubis	pubis	0.409	0.452	0.234	3.68	Null	Probably heated
45	Mai Da Dieu	M7	ulna and radius	rib	0.585	0.617	0.408	2.95	Null	Unheated
46	Mai Da Dieu	M7	ulna and radius	rib	0.0623	0.0709	0.0389	3.42	Null	Probably heated
47	Mai Da Dieu	M8	none	upper limb fragment	0.114	0.129	0.063	3.86	Null	> 400°C
48	Mai Da Dieu	M9a	none	rib	0.287	0.328	0.169	3.64	Null	Probably heated
49	Mai Da Dieu	M10	none	ulna	0.0673	0.0751	0.0411	3.46	Null	Probably heated
50	Mai Da Dieu	M15	none	skull	0.079	0.091	0.042	4.05	Weak	> 400°C
51	Mai Da Dieu	M17	right frontal bone, humerus, ulna, ribs, tibia, and metacarpal bones	metacarpal bone	0.758	0.812	0.227	6.92	Obvious	> 400°C
52	Mai Da Dieu	M17	right frontal bone, humerus, ulna, ribs, tibia, and metacarpal bones	rib	0.153	0.188	0.085	4.01	Weak	> 400°C
53	Mai Da Dieu	M66	none	unknown limb fragment	0.311	0.351	0.196	3.38	Null	Probably heated
54	Con Co Ngua	80CCN-M12E	none	skull	0.232	0.32	0.117	4.72	Null	> 400°C
55	Con Co Ngua	80CCN-M41	distal end of left femur	left femur	0.138	0.148	0.084	3.40	Null	Probably heated
56	Con Co Ngua	80CCN-M44	none	unknown limb fragment	0.502	0.496	0.272	3.67	Null	Probably heated
57	Con Co Ngua	80CCNM44-in_black	humerus	humerus	0.191	0.258	0.109	4.12	Null	> 400°C

**Sample Number**	**Site Name**	**Context**	**Visible black-coloured burned bones of the skeleton**	**Examined bones**	peak/trough intensity			CI	OH^- libration band at ca. 630 cm^{-1}	Group
					peak 603 cm^{-1}	peak 565 cm^{-1}	in-between trouph			
58	Con Co Ngua	80CCNM4 4-out_white	none	skull	0.189	0.197	0.113	3.42	Null	Probably heated
59	Hang Cho	M1	none	skull	0.0638	0.0739	0.0383	3.60	Null	Probably heated
60	Hang Diem	M1	none	rib	0.152	0.185	0.076	4.43	Null	> 400°C
61	Hang Mang Chieng	M5	foot bones	metatarsal bone	0.597	0.604	0.261	4.60	Null	> 400°C
62	Hang Mang Chieng	M2-1	none	skull	0.196	0.221	0.124	3.36	Null	Probably heated
63	Hang Mang Chieng	M2-2	none	unknown limb fragment	0.068	0.075	0.0419	3.41	Null	Probably heated
64	Hang Mang Chieng	M2-3	none	unknown limb fragment	0.11	0.121	0.071	3.25	Null	Probably heated
65	Hang Mang Chieng	M7-in_yellow	fibula (blackened due to heavy smoke? or sediment?)	fibula	0.0798	0.0896	0.0576	2.94	Null	Unheated
66	Hang Mang Chieng	M7-out_black	fibula (blackened due to heavy smoke? or sediment?)	fibula	0.118	0.128	0.079	3.11	Null	Unheated
67	Hang Mang Chieng	M7-out_yellow	fibula (blackened due to heavy smoke? or sediment?)	fibula	0.217	0.245	0.149	3.10	Null	Unheated
68	Gua Harimau	M57	none	skull	0.38	0.457	0.199	4.21	Weak	> 400°C
69	Gua Harimau	M79	none	skull	poor signal					
Control samples (N=1)										
70	Edo Castle	No 1	unknown limbs	unknown limb fragment	0.525	0.59	0.252	4.42	Weak	> 400°C

Table S5. Cut marks observed on human bones from Huiyaotian, Guangxi.

Cut marks were identified on the bones of 19 individuals excavated in 2016 from 18 burial pits at Huiyaotian. This table provides detailed information, including burial number, sex, age, cut mark code, as well as the length and width of each observed cut mark (2).

Burial number	Sex, age	Cutmark code	Maximum length	Maximum width	Skeletal portion
M5	Male, 20-25	M5-H:1	2705.15	307.52	Left humerus, medial supracondylar ridge
		M5-P:1	4766.14	409.36	Left patella, anteromedial surface
			2016.55	265.04	
M9	Male, >40	M9-F:1	1568.65	93.07	Left femur, distal lateral supracondylar line
			1704.37	57.72	
			584.74	—	
			416.7	—	
			270.93	—	
		M9-F:2	2716.14	169.75	Left femur, distal lateral supracondylar line
			812.22	—	
		M9-F:3	3416.93	—	Left femur, distal lateral supracondylar line
			3403.03	—	
			697.73	—	
			2767.38	—	
		M9-F:4	2832.78	64.23	Left femur, distal lateral supracondylar line
			1297.97	339.5	
			2466.78	111.56	
		M9-F:5	3821.48	57.55	Left femur, distal lateral supracondylar line
			4015.64	57.55	
			809.15	47.68	
			3722.86	150.57	
		M9-F:6	2705.75	81.75	Left femur, distal lateral supracondylar line
			3357.86	87.59	
		M9-H:1	2980.92	241.04	Right humerus, lateral supracondylar ridge
		M9-H:2	1629.05	301.02	Right humerus, lateral supracondylar ridge
			1631.56	236.11	
		M9-T:1	1405.28	140.02	Left tibia, anterior border of the shaft
			2599.37	206.44	
			1973.09	172.7	

			1256.16	153.33	
			2317.27	375.1	
M16	Female, 25-30	M16-F:1	2340.55	147.04	Left femur, medial side of the shaft
			3060.05	193.06	
		M16-F:2	4126.55	175.57	Left femur, medial side of the shaft
M21	Male, >50	M21-F:1	2149.5	192.19	Left femur, lateral side of the shaft
			1338.34	53.49	
M35	Female, 25-30	M35-U:1	4776.48	309.30	Right ulna, medial olecranon
			—	243.46	
		M35-U:2	1513.27	169.80	Right ulna, lateral olecranon
		M35-Fi:1	4332.97	257.01	Left fibula, anterior to the lateral malleolus
			1443.12	134.31	
M36	Female, 25±	M36-U:1	1713.13	97.49	Right ulna, medial olecranon
			2510.57	160.34	
M39	Male, 25-30	M39-H:1	9649.76	329.22	Left humerus, medial supracondylar ridge
M40	Male, 30-35	M40-UN:1	3492.32	408.58	Undetermined
M43	Male, 15-20	M43-H:1	2466.82	190.35	Right humerus, superomedial trochlear articular surface
			1244.81	—	
			2006.31	212.71	
M52	Female, 25±	M52-H:1	2518.98	128.37	Right humerus, lateral radial fossa
M53	Male, 40-45	M53-H:1	6457.11	—	Right humerus, medial to the coronoid fossa
			4717.43	—	
		M53-H:2	1349.59	—	Right humerus, medial to the coronoid fossa
			3157.59	—	
		M53-H:3	2081.26	307.26	Right humerus, superior to the coronoid fossa
			3416.6	—	
		M53-H:4	725.79	—	Right humerus, medial epicondyle
			1467.18	140.48	
			2062.14	225.3	
			2231.35	511.73	
		M53-H:5	4295.72	140.02	Right humerus, superolateral to the radial fossa
			9644.19	226.44	
		M53-H:6	5481.47	296.08	Right humerus, radial fossa
M55	Male, 25±	M55-F:1	4291.22	355.08	Left femur, inferoanterior femoral neck
			3511.1	295.4	
			3779.61	315.73	
			1700.54	165.23	
			2527.94	202.99	

			4052.12	299.8	
			3527.16	181.17	
		M55-F:2	5584.13	226.11	Left femur, inferoanterior femoral neck
			1984.4	204.15	
			1990.18	233.04	
M75	Male, >55	M75-H:1	1078.92	148.17	Left humerus, medial margin of the distal end
			980.55	209.3	
M80	Female, 35-40	M80-F:1	2805.35	564.45	Left femur, lateral epicondyle
		M80-F:2	2232.77	326.3	Left femur, lateral epicondyle
			—	207.25	
		M80-F:3	4037.7	445.33	Right femur, superolateral to the patellar surface
		M80-H:1	4697.26	222.72	Left humerus, superior to the coronoid fossa
			2892.36	228.74	
		M80-H:2	1113.36	92.67	Left humerus, superior to the coronoid fossa
			1664.07	122.15	
		M80-T:1	1996.38	164.33	Right tibia, lateral margin of the shaft
M82	Male, old adult	M82-H:1	2708.82	720.33	Right humerus, lateral supracondylar ridge
		M82-U:1	2341.43	202.71	Left ulna, medial olecranon
			1563.08	127.69	
M85	Male, 20±	M85-T:1	2363.84	158.56	Right tibia, anterior border of the shaft
		M85-T:2	2081.9	118.02	Right tibia, anterior border of the shaft
M98	Female, 20-25	M98-F:1	6099.03	301.41	Left femur, inferior femoral neck
			4850.04	228.66	
			3148.95	210.69	
			—	—	
			—	—	
		M98-F:2	1913.91	289.08	Left femur, inferior femoral neck
			2160.8	289.08	
M106-a	Male, 30-40	M106-aH:1	2501.12	447.1	Right humerus, superomedial to the coronoid fossa
		M106-aH:2	1601.57	248.53	Right humerus, lateral supracondylar ridge
M106-b	Unknown, adult	M106-bH:1	1842.55	212.53	Right humerus, lateral supracondylar ridge
		M106-bH:2	823.62	59.87	Right humerus, medial coronoid fossa
			—	272.36	

Supplementary References

1. H. Matsumura, S. Watanabe & M. Yamagata, “Burial practice and individual description of the human skeletal remains at Huiyaotian” in *Bio-Anthropological Studies of Early Holocene Hunter-Gatherer Sites at Huiyaotian and Liyupo in Guangxi, China*, H. Matsumura *et al.*, Eds. (National Museum of Natural Sciences, 2017), pp. 17-60.
2. Y. Zhang, *Preliminary and Related Studies on Cutmarks of Human Bones from Huiyaotian Site, Nanning*. Master’s thesis, Sun Yat-sen University (2020) (in Chinese).
3. IA, CASS, ATGZ, Cultural Relic Survey Team of Guilin City & Zengpiyan Site Museum. *Guilin Zengpiyan* (Cultural Relics, 2003) (in Chinese).
4. P. Shipman, G. Foster & M. Schoenin, Burnt bones and teeth: An experimental study of color, morphology, crystal structure, and shrinkage. *J. Archaeol. Sci.* **11**, 301–325 (1984).
5. H. Matsumura *et al.*, Craniometrics reveal "Two Layers" of prehistoric human dispersal in Eastern Eurasia. *Sci. Rep.* **9**, 1451 (2019).
6. Hunan Antique Archaeology Institute, *Gaomiao site, Hongjiang* (China Sci. Publ. Media Ltd., Beijing, 2022) (in Chinese).
7. X. Fu, Y. Fu, X. Zhang, Z. Zhou, C. Huang, Niupo Cave site, Guian new district, Guizhou. *Kaogu* (*Archaeology*) **7**, 3–17 (2017) (in Chinese).
8. Y. Fu, A preliminary discussion on the relationships between early cave sites in the eastern and western regions of South China. *Cultural Relics in Southern China* **4**, 1–13 (2024) (in Chinese).
9. Fujian Museum, Longyan Bureau of Culture, Radio, Television, Press and Publication, *Qihedong site, Zhangping* (China Sci. Publ. Media Ltd., Beijing, 2017) (in Chinese).
10. C. Y. Chen *et al.*, *Excavation of the sites at the Daowei, Liangdao Island and Liangdao People Restoration Project* (Cultural Bureau, Lianjiang County Gov., 2013) (in Chinese)
11. H. C. Hung *et al.*, Negritos in Taiwan and the wider prehistory of Southeast Asia: New discovery from the Xiaoma Caves. *World Archaeol.* **54**, 1–22 (2022).
12. Guilin Municipal Local Chronicles Compilation Committee, *Guilin Annals* (Zhonghua Book Company, Guilin, 1997) (in Chinese).
13. Y. Jiang, Study of the Bailiandong, Miaoyan and Xianrendong sites – A typical case analysis of the transition from the Palaeolithic to the Neolithic period in South China. *Prehist. Res.* **2006**, 58–67 (2006) (in Chinese).

14. X. Liu, R. Wang, Y. Hu, Carbon and nitrogen stable isotope study of human and animal bones from the Zengpiyan and Dayan sites, Guilin, China. *Kaogu* (*Archaeology*) **7**, 83–95 (2021) (in Chinese).
15. T. Wang *et al.* Prehistoric genomes from Yunnan reveal ancestry related to Tibetans and Austroasiatic speakers. *Science* **388**, eadq9792 (2025).
16. G. Xie, C. Peng, X. Huang, X. Zhou, Archaeological excavations at the Baida site in Bose, Guangxi, made significant discoveries. *China Cultural Relics News* **2006**, 7 April (2006) (in Chinese).
17. Guangxi Institute of Cultural Heritage and Archaeology, Gexinqiao site, Bose. *Cultural Relics Press, Beijing* (2012) (in Chinese).
18. Q. Lin, G. Xie, Y. Ning, Guangxi Du'an Beidaling site archaeological excavations achieve important results. *China Cultural Relics News*, Beijing, p. 1 (2005) (in Chinese).
19. N. He, Y. Huang, W. Liu, Neolithic shell-midden site of Liyuzui, Dalongtan, Liuzhou, China. *Kaogu* (*Archaeology*) **9**, 769–774 (1983) (in Chinese).
20. Y. Fu *et al.*, Preliminary report on the 2023 excavation of the Fengyan site, Liuzhou city, Guangxi. *Kaogu* (*Archaeology*) **2**, 3–18 (2025)(in Chinese).
21. Y. Gu, Y. Li, The Xiankezhou shell-mound site in Longyi, Gaoyao county. *Cultural Relics* **11**, 8–13 (1991) (in Chinese).
22. Guangxi Zhuang Autonomous Region Cultural Relics and Archaeology Training Course, Guangxi Zhuang Autonomous Region Cultural Relics Team, Neolithic shell-midden sites in Guangxi Nanning area. *Kaogu* (*Archaeology*) **5**, 295–301, 326–327 (1975) (in Chinese).
23. Z. Li *et al.*, "Liyupo site in Nanning, Guangxi, China" in *Bio-Anthropological Studies of Early Holocene Hunter-Gatherer Sites at Huiyaotian and Liyupo in Guangxi, China*, H. Matsumura *et al.*, Eds. (National Museum of Natural Sciences, 2017), pp. 95-104.
24. Z. Deng *et al.*, Food and ritual resources in hunter-gatherer societies: Canarium nuts in southern China and beyond. *Antiquity* **93**, 1460–1478 (2019).
25. Z. Hu, A preliminary study of prehistoric shell-midden sites in the Yongjiang River Basin, Guangxi, China. *Civ. Mus. Ser.* **2023**, 24–32 (2023) (in Chinese).
26. Z. Li *et al.*, "Huiyaotian site in Nanning, Guangxi, China" in *Bio-Anthropological Studies of Early Holocene Hunter-Gatherer Sites at Huiyaotian and Liyupo in Guangxi, China*, H. Matsumura *et al.*, Eds. (National Museum of Natural Sciences, 2017), pp. 7-20.

27. X. Fu, X. Li, Z. Li, L. Zhang, C. Chen, Excavations at the Dingsishan site, Yongning county, Guangxi, China. *Kaogu* (*Archaeology*) **11**, 11–33 (1998) (in Chinese).
28. Q. Liu, Preliminary analysis of prehistoric cemeteries in Qiujiang, Guangxi. Master's thesis, Minzu University of China (2012) (in Chinese).
29. S. Peng, T. Jiang, The Xijin shell-midden site and its shouldered stone tools, Guangxi, China. *Southeast Culture,* Supplementary Issue **1**, 164–172 (1991) (in Chinese).
30. A. He, Q. Yang, Y. Ning, New archaeological discoveries and preliminary understanding of shell-midden sites in the Zuojiang Basin, Guangxi. *Chin. Hist. Antiq.* **5**, 4–10 (2009) (in Chinese).
31. H. Mansuy, M. Colani, Contribution à l'étude de la préhistoire de l'Indochine VII: Néolithique inférieur (Bacsonien) et Néolithique supérieur dans le Haut-Tonkin. *Bulletin du Service Géologique de l'Indochine* **12**, 1–45 (1925) (in French).
32. L. C. Nguyen, *Vietnamese Physical Anthropology: Scientific Evidence for Understanding the Origins of the Vietnamese People* (Institute of Archaeology, Vietnam Academy of Social Sciences, Hanoi, 2011) (in Vietnamese).
33. L. C. Nguyen, K. D. Nguyen, H. D. Le, *Excavation Report on Hòn Hai Cô Tiên (Hòn Cặp Bè), Bạch Đằng Ward, Hạ Long City (Quảng Ninh)- Second Excavation, 2021* (Vietnam Archaeology Association, Hanoi, 2022) (in Vietnamese).
34. K. S. Nguyen, *Prehistorical Site Cai Beo on Cat Ba Island* (Social Science, Hanoi, 2009) (in Vietnamese).
35. F. Li, H. Chen, C. Zou, B. Yang, M. Feng, Excavation brief of the Neolithic shell-mound site of Liyudun in Suixi, Guangdong province, China. *Cultural Relics* **7**, 4–18 (2015) (in Chinese).
36. L. C. Nguyen, "Austronesian and Australian analogs in Vietnam through paleoanthropological evidence" in *Austronesian Diaspora: A New Perspective*, B. Prasetyo, T. S. Nastiti, T. Simanjuntak, Eds. (Gadjah Mada University Press, 2016), pp. 145-163.
37. V. Nguyen, "First archaeological evidence of symbolic activities from the Pleistocene of Vietnam" in *Emergence and Diversity of Modern Human Behavior in Paleolithic Asia*, Y. Kaifu, M. Izuho, T. Goebel, H. Sato, A. Ono, Eds. (Texas A&M Univ. Press, College Station, 2015), pp. 133–139.

38. M. Colani, La grotte sépulcrale de Lang Gao, *L'Anthropologie* **37**, 227–229 (1927)(in French).
39. H. Matsumura *et al.*, Terminal Pleistocene human skeleton from Hang Cho Cave, northern Vietnam: Implications for the biological affinities of Hoabinhian people. *Anthropol. Sci.* **116**, 201–217 (2008).
40. D. Bulbeck, M. Oxenham & L. C. Nguyen, Implications of the Terminal Pleistocene skull from Hang Muoi, North Vietnam. *Khao Co Hoc* **2**, 42–52 (2007).
41. V. Nguyen, H. N. Ha, K. D. Nguyen, Test excavation at Xom Trai. *New Discov. Archaeol.* **1982**, 43–46 (1982) (in Vietnamese).
42. C. McAdams *et al.*, Late Pleistocene shell midden microstratigraphy indicates a complex history of human-environment interactions in the uplands of northern Vietnam. *Phil. Trans. R. Soc. B* **377**, 20200493 (2022).
43. A. P. Derevianko *et al*., Results of field research of Mang Chieng Cave (Vietnam) in 2011. *Probl. Archaeol. Ethnogr. Anthropol. Siberia Neighbor. Territ.* **18**, 59–62 (2012).
44. A. P. Derevianko *et al*., Results of field research of Mang Chieng Cave (Vietnam) in 2012. *Probl. Archaeol. Ethnogr. Anthropol. Siberia Neighbor. Territ.* **29**, 66–69 (2013).
45. H. T. Pham, Con Moong Cave: A noteworthy archaeological discovery in Vietnam. *Asian Perspect.* **23**, 17–21 (1980).
46. H. D. Le, A. T. Nguyen, A. Kandyba, Excavation at Hang Diem (Thanh Hoa) in 2013. *Khao Co Hoc* (*Vietnam Archaeol.*) **1**, 39–47 (2015)(in Vietnamese).
47. V. B. Nguyen, Preliminary report on the 2nd excavation at Dieu Rockshelter. *Khao Co Hoc* (*Vietnam Archaeol.*) **3**, 24-34 (1994) (in Vietnamese).
48. L. C. Nguyen, Two early Hoabinhian crania from Thanh Hoa Province, Vietnam. *Z. Morphol. Anthropol.* **77**, 11–17 (1986).
49. M. Colani, *L'Âge de la Pierre dans la Province de Hòa Bình*, *Mémoires du Service Géologique de l'Indochine* **13**, 1–86 (1927) (in French).
50. M. Colani, La civilisation Hoabinhienne extrême-orientale, *Bulletin de la Société Préhistorique Française* **36**, 170–174 (1939) (in French).
51. V. Nguyen, The Da But Culture: evidence for cultural development in Vietnam during the Middle Holocene. *Bull. Indo-Pac. Prehist. Assoc.* **25**, 89–94 (2007).

52. M. F. Oxenham *et al.*, Between foraging and farming: Strategic responses to the Holocene Thermal Maximum in Southeast Asia. *Antiquity* **92**, 940–957 (2018).

53. T. E. G. Reynolds, Excavations at Banyan Valley Cave, northern Thailand: A report on the 1972 season. *Asian Perspect.* **31**, 77–97 (1992).

54. C. F. Gorman, Excavations at Spirit Cave, north Thailand: Some interim interpretations. *Asian Perspect.* **13**, 79 (1970).

55. T. Chitkament, C. Gaillard, R. Shoocongdej, Tham Lod rockshelter (Pang Mapha district, north-western Thailand): Evolution of the lithic assemblages during the Late Pleistocene. *Quat. Int.* **416**, 151–161 (2016).

56. C. Treerayapiwat, Patterns of habitation and burial activity in the Ban Rai Rock Shelter, northwestern Thailand. *Asian Perspect.* **44**, 231–245 (2005).

57. S. Imdirakphol *et al.*, The perforated stones of the Doi Pha Kan burials (Northern Thailand): A Mesolithic singularity? *C. R. Palevol* **16**, 351–361 (2017).

58. V. Zeitoun *et al.*, Discovery of a Mesolithic burial near the painted rock-shelter of Ban Tha Si (Lampang province, Northern Thailand): Implications for regional mortuary practices. *C. R. Palevol* **12**, 127–136 (2013).

59. V. T. Ha, Nouvelles recherches préhistoriques et protohistoriques au Vietnam. *Bulletin de l'École française d'Extrême-Orient* **68**, 113-154 (1980) (in French).

60. N. Tayles, S. E. Halcrow, T. Sayavongkhamdy, V. Souksavatdy, A prehistoric flexed human burial from Pha Phen, Middle Mekong Valley, Laos: its context in Southeast Asia. *Anthropol. Sci.* **123**, 1–12 (2015).

61. X. Fu *et al.*, Neolithic remains in the coastal areas of southeastern Hainan. *Kaogu* (*Archaeology*) **7**, 3–18 (2016) (in Chinese).

62. L. C. Nguyen, The ancient human remains in Bau Du (Quang Nam) excavated in 2014. *Khao Co Hoc* **2**, 3–16 (2016) (in Vietnamese).

63. V. T. Ha, Différentes lignes de développement du post-Hoabinhien à l'Âge de la Pierre au Vietnam. *L'Anthropol.* **99**, 652–656 (1995) (in French).

64. V. T. Ha, Different lines of post-Hoabinhian cultural development in the Stone Age in Vietnam. *Vietnam Soc. Sci.* **2**(52), 24–41 (1996).

65. K. S. Nguyen *et al*., Excavation at volcanic cave C6-1 Krong No (Dak Nong): new data and perception of Tay Nguyen's prehistory. *Khao Co Hoc* (*Vietnam Archaeol.*) **226**, 16-30 (2020) (in Vietnamese).
66. A. Pawlik, R. Crozier, R. Fuentes, R. Wood, P. Piper, Burial traditions in early Mid-Holocene Island Southeast Asia: New evidence from Bubog-1, Ilin Island, Mindoro Occidental. *Antiquity* **93**, 901–918 (2019).
67. M. Lara *et al*., Bone modifications in an Early Holocene cremation burial from Palawan, Philippines. *Int. J. Osteoarchaeol.* **25**, 637–652 (2015).
68. R. B. Fox, *The Tabon Caves: Archaeological Explorations and Excavations on Palawan Island, Philippines* (Natl. Mus. Philippines, Manila, 1970).
69. J. H. Kress, "The necrology of Sa'gung rockshelter and its place in Philippine prehistory" in *Southeast Asian Archaeology*, V. Paz, Ed. (Univ. Philippines Press, Quezon City, 2004), pp. 239–275.
70. P. Auetrakulvit, H. Forestier, C. Khaokhiew, V. Zeitoun, "New excavation at Moh Khiew site, southern Thailand" in *Crossing Borders*, M. L. Tjoa-Bonatz, A. Reinecke, D. Bonatz, Eds. (NUS Press, Singapore, 2012), pp. 60–70.
71. P. Van Stein Callenfels, An excavation of three kitchen middens at Guak Kepah, Province Wellesley. *Bull. Raffles Mus. Ser. B* **1**(1), 27–37 (1936).
72. M. Zuraina, *The Perak Man and Other Prehistoric Skeletons of Malaysia* (Penerbit Univ. Sains Malaysia, Penang, 2005).
73. H. M. Goh, M. Saidin. The prehistoric human presence in Gua Kajang: Ancient lifeways in the Malay Peninsula. *J. Malaysian Branch R. Asiat. Soc.* **91**, 1–18 (2018).
74. B. Harrisson, A classification of Stone Age burials from Niah Great Cave, Sarawak. *Sarawak Mus. J.* **15**, 126–200 (1967).
75. G. Barker *et al.*, Foraging–farming transitions at the Niah Caves, Sarawak, Borneo. *Antiquity* **85**, 492–509 (2011).
76. Harrisson, B. *Field notebook Burial B147* (Sarawak Museum, n.d.).
77. L. Lloyd-Smith, Early Holocene burial practice at Niah Cave, Sarawak. *J. Indo-Pac. Archaeol.* **32**, 54–69 (2012).

78. K. Arifin, “Terminal Pleistocene and Early Holocene human occupation in the rainforests of East Kalimantan” in *New Perspectives in Southeast Asian and Pacific Prehistory*, P. J. Piper, H. Matsumura, D. Bulbeck, Eds. (ANU Press, Canberra, 2017), pp. 97–124.
79. T. R. Maloney *et al.*, Surgical amputation of a limb 31,000 years ago in Borneo. *Nature* **609**, 547–551 (2022).
80. J.-M. Chazine, J.-G. Ferrie, Recent archaeological discoveries in East Kalimantan, Indonesia. *Bull. Indo-Pac. Prehist. Assoc.* **28**, 16–22 (2008).
81. N. Fajari, V. Kusmartono, The excavation of Gua Payung, south Kalimantan, Indonesia. *Bull. Indo-Pac. Prehist. Assoc.* **33**, 20–23 (2014).
82. S. Carlhoff *et al.*, Genome of a Middle Holocene hunter-gatherer from Wallacea. *Nature* **596**, 543–547 (2021).
83. S. Suryatman *et al.*, Incised stone artefact in the context of Middle Holocene burials at Cappalombo 1, South Sulawesi, Indonesia. *SPAFA J.* **5**, 1–23 (2021).
84. M. R. Fauzi *et al.*, The excavation of Gua Harimau's western gallery: A contribution to the Terminal Pleistocene-Early Holocene archaeological records in Sumatra. *L'Anthropologie* **127**, 1–19 (2023).
85. T. Simanjuntak (Ed.), *Harimua Cave and the Long Journey of OKU Civilization* (Gadjah Mada University Press, Yogyakarta, 2016).
86. M. R. Fauzi, A. Oktaviana, Budiman, “Jejak budaya paleometalik dan kronologinya di Gua Harimau” in *Perjalanan Panjang Peradaban OKU*, T. Simanjuntak, Ed. (UGM Press, Yogyakarta, 2015), pp. 138–147 (In Bahasa).
87. H. Matsumura *et al.*, Cranio-morphometric and aDNA corroboration of the Austronesian dispersal model in ancient Island Southeast Asia: Support from Gua Harimau, Indonesia. *PLOS ONE* **13**, e0198689 (2018).
88. S. Noerwidi, “Using dental metrical analysis to determine the Terminal Pleistocene and Holocene population history of Java” in *New Perspectives in Southeast Asian and Pacific Prehistory*, P. J. Piper, H. Matsumura, D. Bulbeck, Eds. (ANU Press, Canberra, 2017), pp. 79–96.
89. T. Ingicco *et al.*, From food to grave good nonhuman primate exploitation in Early to Mid-Holocene eastern Java (Indonesia). *Current Anthropology* **61**, 264–277 (2020).

90. T. Simanjuntak, New light on the prehistory of the Southern Mountains of Java. *Bull. Indo-Pac. Prehist. Assoc.* **5**(21), 152–156 (2001).
91. T. Simanjuntak, New insight on the prehistoric chronology of Gunung Sewu, Java, Indonesia. *Mod. Quat. Res. Southeast Asia* **18**, 9–30 (2004).
92. F. Détroit. "Homo sapiens in Southeast Asian archipelagos: The Holocene fossil evidence with special reference to funerary practices in East Java" in *Austronesian Diaspora and the Ethnogeneses of People in Indonesian Archipelago*, T. Simanjuntak, L. Pojoh, M. Hisyam, Eds. (Indonesian Institute of Sciences, Jakarta, 2006), pp. 186-204.
93. T. Simanjuntak, I. Asikin, Early Holocene human settlement in eastern Java. *Indo-Pacific Prehistory Association Bulletin* **2**, 13–19 (2004).
94. S. C. Samper-Carro *et al.*, Talking dead: New burials from Tron Bon Lei (Alor Island, Indonesia) inform on the evolution of mortuary practices from the Terminal Pleistocene to the Holocene in Southeast Asia. *PLOS ONE* **17**, e0267635 (2022).
95. S. Hawkins *et al*., Earliest known funerary rites in Wallacea after the last glacial maximum. *Sci. Rep.* **14**, 282 (2024).
96. D. Bulbeck, "The last glacial maximum human burial from Liang Lemdubu in northern Sahulland" in *The Archaeology of the Aru Islands, Eastern Indonesia*, S. O'Connor, M. Spriggs, P. M. Veth, Eds. (ANU Press, Canberra, 2005), pp. 255–294.
97. H. McColl *et al*., The prehistoric peopling of Southeast Asia. *Science* **361**, 88–92 (2018).
